# Cooperative definition of ER exit sites by Tango1, Sec16 and Sec12

**DOI:** 10.1242/jcs.264950

**Published:** 2026-09-29

**Authors:** Zhi Feng, Mercedes Martín, Marta Rojas, José Carlos Pastor-Pareja

**Affiliations:** ^1^ School of Life Sciences, Tsinghua University, Beijing 100084, China; ^2^ Institute of Neurosciences, Consejo Superior de Investigaciones Científicas-Universidad Miguel Hernández, San Juan de Alicante 03550, Spain

**Keywords:** ER exit site, Golgi, Secretory pathway, Traffic, *Drosophila*

## Abstract

Endoplasmic reticulum (ER) exit sites (ERES) are dynamic ER subdomains where secretory cargos are concentrated and exported towards the Golgi. The conserved proteins Sec16 and Sec12 have long been implicated in ERES organization, whereas animal-specific Tango1 has more recently emerged as a possible additional determinant. Their respective roles in ERES morphogenesis, however, remain unclear. Using *Drosophila*, we dissect the mutual requirements and specific functions of Sec16, Sec12 and Tango1 in defining ERES. We show that Sec16 and Tango1 are interdependent for proper ERES assembly, with co-overexpression producing exaggerated ERES structures. We further identify CG9175 as the *Drosophila* ortholog of Sec12 and demonstrate its upstream requirement for coordinated concentration of Tango1 and Sec16. Tango1 is in turn important for ERES–Golgi coupling. Finally, both Tango1 and Sec16 are required for concentration of COPII and COPI components, albeit with different relative impacts. Together, our findings support the evolutionary incorporation of Tango1 into a cooperative network in which Sec12, Sec16 and Tango1 make distinct contributions to the definition of animal ERES.

## Teaser

**Summary:** During the morphogenesis of ER exit sites in *Drosophila*, Tango1, Sec16 and Sec12 cooperate hierarchically to establish ER exit sites, with each protein making both overlapping and distinct functional contributions.

## INTRODUCTION

The secretory pathway is a complex endomembrane network that coordinates the synthesis, modification and delivery of proteins and lipids to their correct intracellular and extracellular destinations (Barlowe and Miller, 2013; Palade, 1975). By driving traffic between the endoplasmic reticulum (ER), the Golgi and post-Golgi compartments, the secretory pathway supports membrane homeostasis, cellular architecture and intercellular communication. At the starting point of the pathway are ER exit sites (ERES), specialized ER subdomains where secretory cargo is concentrated and packaged for export to the Golgi (Bannykh et al., 1996). ERES function as spatially defined regions that orchestrate membrane curvature, carrier formation and cargo selection (Zanetti et al., 2012). In addition to mediating anterograde export, ERES also serve as ER arrival sites for retrograde carriers that return escaped ER residents and recycle trafficking machinery (Brandizzi and Barlowe, 2013; Langhans et al., 2012). Importantly, ERES are not static structures, but dynamic platforms that must preserve their identity despite continuous membrane flux and rapid protein turnover at the ER-Golgi interface (Farhan et al., 2025). How ERES are specified, assembled and maintained as persistent yet adaptable functional units remains a central question in cell biology. Elucidating the molecular interactions that define ERES identity is essential for understanding how cells achieve efficient secretion while preserving compartmental organization.

ERES are enriched sites of concentration for many proteins, including COPII and COPI coat components, cargo receptors, regulatory GTPases, tethers, fission and fusion regulators, and scaffolding proteins. Although a vast array of factors transiently associate with or stably accumulate at these sites, only a select subset of these is thought to play a decisive role in establishing and maintaining spatial ERES identity. Among these, Sec16 has long been recognized as a key organizer of ERES. First identified through genetic screens in the yeast *Saccharomyces cerevisiae* (Kaiser and Schekman, 1990), Sec16 is a large cytoplasmic protein that associates with the ERES surface. It has been shown to stabilize nascent COPII assemblies and spatially restrict coat formation, thereby ensuring the maintenance of discrete, functional export sites (Connerly et al., 2005; Espenshade et al., 1995; Sprangers and Rabouille, 2015). Another central factor in ERES definition is Sec12, also identified in yeast (Nakano et al., 1988). Sec12 is an ER-resident transmembrane protein characterized by a luminal N-terminus and cytosolic WD40-repeat domains. In contrast with the tight concentration of Sec16 at ERES, Sec12 is more generally distributed throughout the ER (Soderholm et al., 2004). Sec12 has been shown to act as a guanine nucleotide exchange factor for Sar1 in the initiation of COPII coat assembly (Barlowe and Schekman, 1993). Together, conserved Sec16 and Sec12, which have been shown to physically interact (Montegna et al., 2012), are widely considered foundational determinants of ERES formation.

More recently, evidence has pointed to Tango1, a protein present only in animals, as a regulator of ERES organization in addition to Sec16 and Sec12. Tango1 is an ERES-localized transmembrane protein characterized by a lumenal SH3-like domain and a cytoplasmic C-terminal region containing coiled-coil and proline-rich domains (Saito et al., 2009). Although initially identified as a receptor for bulky cargo like collagen, studies in *Drosophila melanogaster* have shown that Tango1 is present at all ERES and is required for preserving ERES integrity and supporting general secretion (Liu et al., 2017; Ríos-Barrera et al., 2017). These findings argue that Tango1 is a core scaffolding component of animal ERES rather than a specialized cargo receptor or adaptor. Furthermore, biochemical analyses have shown that Tango1 interacts with a vast array of generally required secretion factors, including COPII (Saito et al., 2009), SNAREs (Nogueira et al., 2014; Santos et al., 2015), Rab1, GRASP65 (Liu et al., 2017) and p24 proteins (Yang et al., 2024). Notably, Tango1 also physically associates with both Sec16 and Sec12 (Maeda et al., 2017; Saito et al., 2014). However, unlike Sec16 and Sec12, Tango1 is a metazoan-specific evolutionary innovation, raising questions on how Tango1 functionally integrates with the more ancient Sec16 and Sec12. Moreover, the extent to which each of these proteins contribute uniquely to animal ERES identity is unclear.

The fruit fly *Drosophila melanogaster* provides an excellent system to address secretory pathway architecture and ERES morphogenesis in the context of an intact animal (Kondylis and Rabouille, 2009). In *Drosophila*, the early secretory pathway is organized into discrete ERES-Golgi units where individual ERES are stably apposed to Golgi elements. A major advantage of *Drosophila* is the high degree of homology of the core secretory machinery, including the COPII and COPI coat systems, SNAREs, Rab GTPases, TRAPP tethering complexes and p24 proteins, which are all well conserved and functionally analogous to their mammalian, yeast or plant counterparts (Bard et al., 2006; Ke et al., 2018; Wendler et al., 2010). Furthermore, both vesicular and tubular carriers have been described in fly ERES (Yang et al., 2021), as in human cells (Nair et al., 2026), underscoring its relevance for studying diverse modes of ER–Golgi transport. Regarding the regulation of ERES morphogenesis, importantly, *Drosophila* contains single homologs of the key ERES-defining factors. *Drosophila* encodes a single Sec16 homolog (Ivan et al., 2008), in contrast to the two paralogs SEC16A and SEC16B present in humans (Bhattacharyya and Glick, 2007; Watson et al., 2006). There is, in addition, only one Tango1-encoding gene in *Drosophila*, producing a single protein isoform, whereas mammals possess 10 paralog genes encoding at least 15 different Tango1 and Tango1-like proteins with partially overlapping functions (Feng et al., 2021; Saito et al., 2011; Santos et al., 2016). Finally, there is a single putative SEC12 ortholog in flies, but it has not been functionally characterized, leaving a key gap in our understanding of ERES regulation in this model. Notably, SEC12 functions in *S. cerevisiae* are shared by Sec12 and paralog Sed4 (Yorimitsu and Sato, 2023). Reduced genetic redundancy in *Drosophila*, therefore, might greatly simplify functional analysis.

In this study, we used *Drosophila* as an *in vivo* system to dissect the mutual requirements and specific roles of Sec12, Sec16 and Tango1 in ERES organization. We functionally characterized the *Drosophila* Sec12 ortholog and demonstrate that Sec12, Sec16 and Tango1 are all required to generate functional ERES. Our analyses reveal that these proteins act cooperatively yet fulfill distinct roles in ERES morphogenesis, coatomer recruitment and ER-Golgi coupling, providing a hierarchical framework for understanding how dynamic ERES are defined and maintained.

## RESULTS

### Mutual recruitment of Tango1 and Sec16 to ERES

Widely conserved Sec16 and metazoan-specific Tango1 are proteins that highly concentrate at ERES and contribute to ERES organization. To clarify their relation and respective roles in ERES (Fig. 1A), we investigated their functions in *Drosophila*, which possesses single Sec16 and Tango1 homologs. To do that, we first examined their mutual effects upon overexpression in the adipocytes of the fat body, a highly secretory tissue that produces large amounts of extracellular matrix and other proteins present in the hemolymph of the third-instar larva (Pastor-Pareja and Xu, 2011). We overexpressed Sec16 or Tango1 in the fat body through the GAL4-UAS system (Brand and Perrimon, 1993), using the fat body-specific driver Cg-GAL4. Localization of Tango1 and Sec16 was analyzed by confocal microscopy using superfolder GFP (sGFP)-tagged constructs expressed under their endogenous promoters (Sarov et al., 2016). Sec16 overexpression (Sec16 OE) led to the formation of abnormally large Tango1 puncta compared with the punctate pattern observed in the wild type (Fig. 1B). Conversely, Tango1 overexpression (Tango1 OE) resulted in enlarged Sec16 puncta (Fig. 1C). These results indicate that Sec16 and Tango1 mutually influence each other's localization to ERES. Moreover, although overexpression of Sec16 or Tango1 were separately capable of inducing large ERES puncta, their combined overexpression produced a noticeable increase in puncta abundance and generated some strikingly large circular structures in which both proteins colocalized (Fig. 1D–F). Quantitative analysis of these puncta in confocal images showed that overexpression of either Sec16 or Tango1 separately increased puncta abundance and size significantly, and that these effects were further exacerbated upon co-overexpression (Fig. 1G,H). Together, these results support coordinated mutual recruitment of Sec16 and Tango1 to *Drosophila* ERES.

**Fig. 1.**
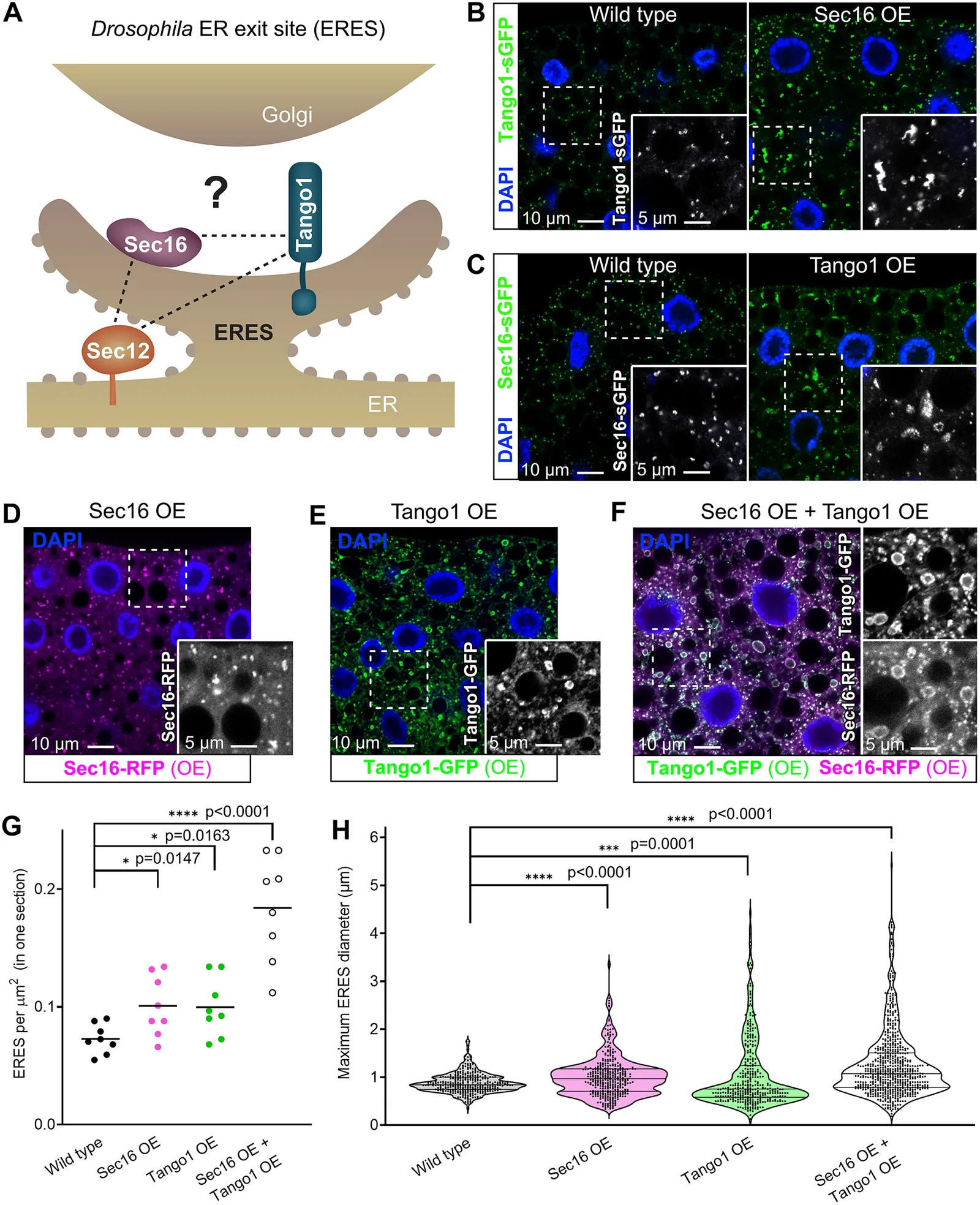
**Mutual recruitment of Tango1 and Sec16 to ERES.** (A) Schematic representation of an ERES–Golgi unit in *Drosophila* displaying proteins Tango1, Sec16 and Sec12 on a cup-shaped ERES. (B) Tango1 distribution (Tango1–sGFP, green) in adipocytes of wild-type (left) and Sec16-overexpressing (Sec16 OE, right) fat body from third-instar larvae. Nuclei stained with DAPI (blue). Images in bottom right corners (Tango1–sGFP, white) show magnified views of areas highlighted by dashed squares. (C) Sec16 distribution (Sec16–sGFP, green) in adipocytes of wild-type (left) and Tango1-overexpressing (Tango1 OE, right) fat body. Nuclei stained with DAPI (blue). Images in bottom right corners (Sec16–sGFP, white) show magnified views of insets (dashed squares). (D) Distribution of overexpressed Sec16 (UAS-Sec16–RFP, magenta) in fat body. Image in bottom right corner (Sec16–RFP, white) shows a magnified view of the area highlighted by a dashed square. (E) Distribution of overexpressed Tango1 (UAS-Tango1–GFP, green) in fat body. Image in the bottom right corner (Tango1-GFP, white) shows a magnified view of the area highlighted by a dashed square. (F) Distribution of co-overexpressed Sec16 (UAS-Sec16–RFP, magenta) and Tango1 (UAS-Tango1–GFP, green) in fat body. Images on the top right (Tango1–GFP, white) and bottom right (Sec16–RFP, white) show magnified views of inset areas highlighted by dashed squares. (G) Graph comparing ERES abundance in wild-type, Sec16-overexpressing, Tango1-overexpressing and Sec16+Tango1-co-overexpressing fat body. Each dot represents a measurement of the density of Sec16 or Tango1 puncta counted in a square of the dimensions of the insets in B–F. The mean is highlighted; *n=*8 images per genotype. (H) Graph comparing ERES size in wild-type, Sec16-overexpressing, Tango1-overexpressing and Sec16+Tango1-co-overexpressing fat body adipocytes. Each dot represents a measurement of the maximum length of a Sec16 or Tango1 punctum. Violin plots are shown with median value and interquartile range marked; *n*≥326 puncta per genotype. ****P<0.0001; ***P<0.001; *P<0.05 for means compared with wild type (two-tailed unpaired *t*-tests).

### Loss of Tango1 or Sec16 causes reduction of ERES size and number

To directly interrogate and compare the roles of Sec16 and Tango1 in ERES organization, we next analyzed loss-of-function conditions for both. To this end, we individually knocked down the expression of each protein using GAL4-driven UAS-RNAi transgenes under control of fat body-specific driver Cg-GAL4 and analyzed the effect on the localization of the other protein using again sGFP-tagged versions of Tango1 and Sec16 expressed under their endogenous promoters. Sec16 depletion through knockdown (*Sec16^i^*) led to a marked reduction in the number and size of Tango1-positive puncta, which appeared smaller than in the wild type (Fig. 2A). Similarly, knockdown of Tango1 (*Tango1^i^*) led to a pronounced decrease in the number and size of Sec16 puncta (Fig. 2B). Depletion of Sec16 and Tango1 in these knockdown conditions were highly efficient, as sGFP fluorescence and protein levels of the targeted proteins were undetectable for both Sec16 in *Sec16^i^* fat body (Fig. 2C,D) and Tango1 in *Tango1^i^* fat body (Fig. 2E,F). Quantitative image analysis confirmed the phenotypic effects in puncta size and number, revealing significant reductions for these parameters in both *Sec16^i^* and *Tango1^i^* conditions relative to wild-type controls (Fig. 2G–J). These loss of function experiments show that Sec16 and Tango1 are both required for correct ERES morphogenesis. To additionally test whether Tango1 and Sec16 are required to form ERES, we conducted experiments in which we knocked down the expression of Tango1 or Sec16 while overexpressing the other protein: *Sec16^i^*+Tango1 OE (Fig. S1A,B) and *Tango1^i^*+Sec16 OE (Fig. S1C,D). In both cases, we observed a reduction in the number of ERES (Fig. S1E) accompanied by an increase in average puncta size (Fig. S1F), indicating phenotypic prevalence of the knockdown and overexpression conditions on ERES number and ERES size, respectively. Altogether, both loss and gain of function results support a model in which Sec16 and Tango1 function in a coordinated and interdependent manner to define and maintain ERES.

**Fig. 2.**
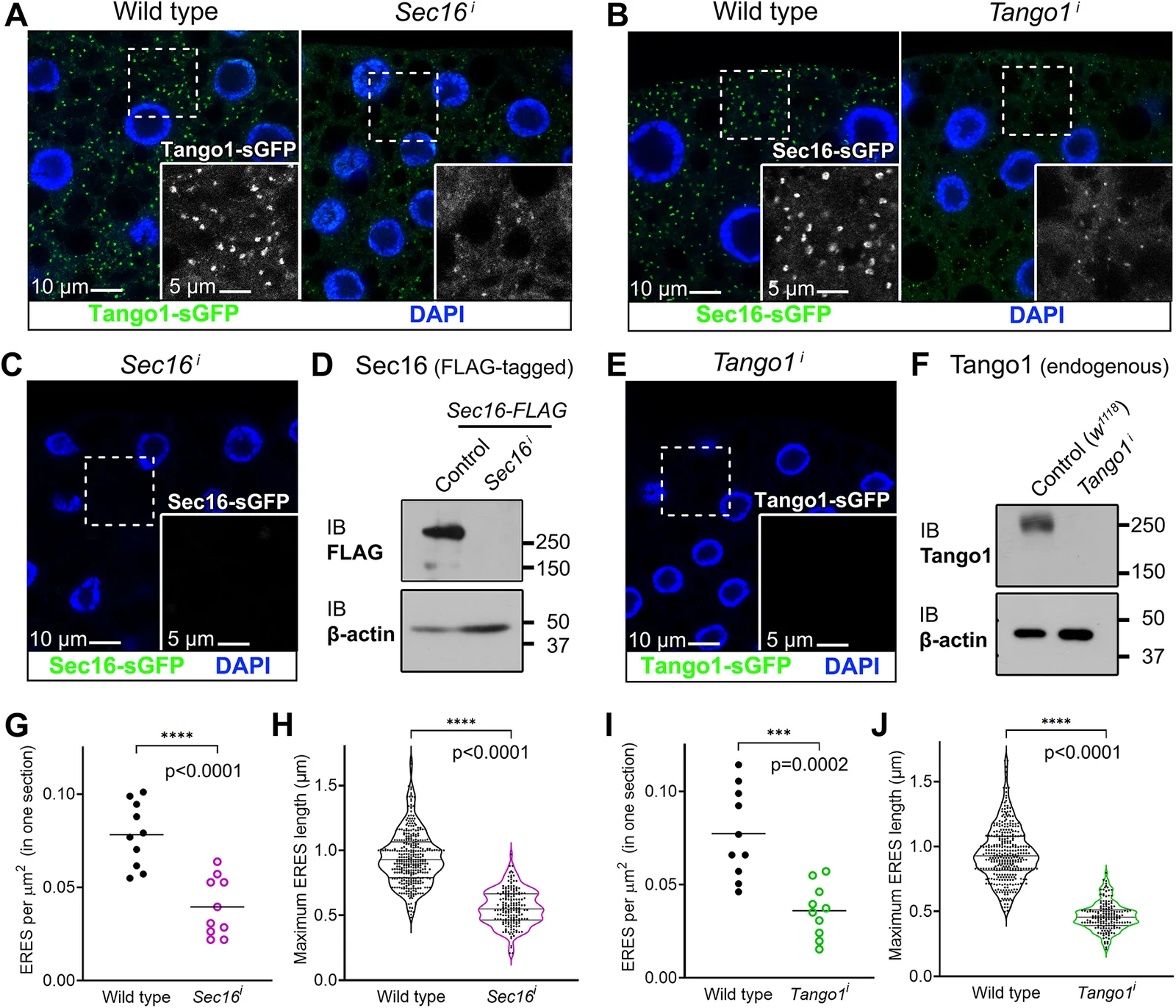
**Loss of Tango1 or Sec16 causes reduction of ERES size.** (A) Tango1 distribution (Tango1–sGFP, green) in adipocytes of wild-type fat body (left) and fat body where Sec16 expression has been knocked down (*Sec16^i^*, right). Nuclei stained with DAPI (blue). Images in the bottom right corners (Tango1–sGFP, white) show magnified views of areas highlighted by dashed squares. (B) Sec16 distribution (Sec16–sGFP, green) in adipocytes of wild-type fat body (left) and fat body where Tango1 expression has been knocked down (*Tango1^i^*, right). Nuclei stained with DAPI (blue). Images in the bottom right corners (Tango1-sGFP, white) show magnified views of areas highlighted by dashed squares. (C) Distribution of Sec16 (Sec16–sGFP, green) in *Sec16^i^* fat body. This is lower compared to the wild-type level in B. Image in the bottom right corner (Sec16-sGFP, white) shows magnified view of the area highlighted by the dashed square. (D) Wild-type control (left) and *Sec16^i^* (right) fat body lysates immunoblotted (IB) with anti-FLAG antibody (top) to detect Sec16–FLAG expressed in both under control of endogenous promoter and anti-β-actin antibody (bottom) as a loading control. (E) Distribution of Tango1 (Tango1–sGFP, green) in *Tango1^i^* fat body. This is lower compared to the wild-type level in A. Image in the bottom right corner (Tango1–sGFP, white) shows magnified view of the area highlighted by the dashed square. (F) Wild-type control (left) and *Tango1^i^* (right) fat body lysates immunoblotted (IB) with anti-Tango1 antibody (top) and anti-β-actin antibody (bottom) as a loading control. Blots in D and E are representative of three experimental repeats. (G) Graph comparing ERES abundance in wild-type and *Sec16^i^* fat body adipocytes. Each dot represents a measurement of the density of Tango1 puncta counted inside a square of the dimensions of the insets in C and E. The mean is highlighted; *n*=10. (H) Graph comparing ERES size in wild-type and *Sec16^i^* fat body adipocytes. Each dot represents a measurement of the maximum length of a Tango1 punctum. Violin plots are shown with median value and interquartile range marked; *n*≥180. (I) Graph comparing ERES abundance in wild type and *Tango1^i^* fat body adipocytes. Each dot represents a measurement of the number of Sec16 puncta in one fat body cell counted in a single confocal image. The mean is highlighted; *n*=10. (J) Graph comparing ERES size in wild-type and *Tango1^i^* fat body adipocytes. Each dot represents a measurement of the maximum length of a Sec16 punctum. Violin plots are shown with median value and interquartile range marked; *n*≥164 puncta per genotype. ****P<0.0001; ***P<0.001 for means compared with wild type (two-tailed unpaired *t*-tests).

### CG9175 encodes *Drosophila* Sec12

In addition to Sec16 and Tango1, Sec12 has also been implicated in ERES definition. However, a Sec12 homolog in *Drosophila* has not been functionally characterized. We therefore investigated *CG9175* (*Coding Gene 9175*; https://flybase.org/reports/FBgn0031779), located on the left arm of the second chromosome (2L), which encodes a single protein that exhibits high sequence identity and similarity to human SEC12 (also known as PREB) and to *S. cerevisiae* SEC12 (Fig. 3A). Moreover, the protein encoded by *CG9175* displays a conserved domain organization, including several WD40 repeat domains and a predicted transmembrane domain at the C-terminus, consistent with known Sec12 proteins (Fig. 3B). Expression of a CFP-tagged version of this protein under control of fat body driver Cg-GAL4 revealed a wide cellular distribution overlapping with the ER marker Sec61β–tdTom (Fig. 3C), consistent with reports of ER localization for Sec12 in other organisms (Montegna et al., 2012; Nishikawa and Nakano, 1993). We additionally examined the localization of this protein in wing disc cells, which are capable of undergoing mitosis, unlike fat body cells, which are polyploid endocycling cells. In dividing wing cells, we found that Tango1 loses its punctate localization and redistributes to the general ER (Fig. S2A), consistent with reports on human Tango1 (Maeda et al., 2025). The protein encoded by *CG9175* showed a similar distribution to Tango1 (Fig. S2B). Sec16 puncta in turn disappeared (Fig. S2C), suggesting cytoplasmic dispersion, whereas Golgin245, a trans-Golgi protein, showed punctate distribution (Fig. S2D), pointing to Golgi persistence during the mitotic cycle. Altogether, sequence conservation, domain organization and subcellular localization support that the *Drosophila* ortholog of Sec12 is encoded by *CG9175*, and hence this is hereafter designated *Sec12*.

**Fig. 3.**
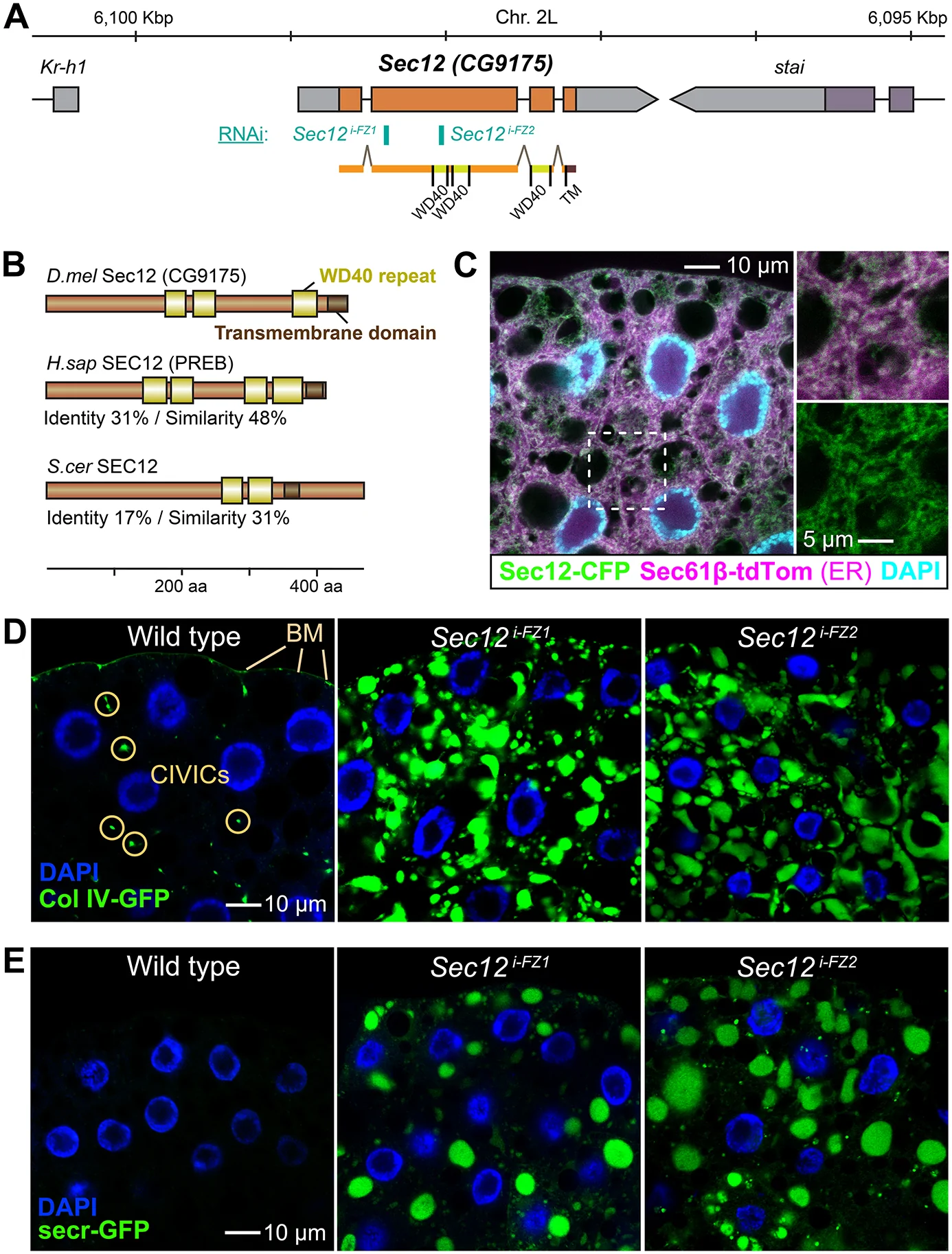
***Drosophila* Sec12 encoded by *CG9175* is required for secretion.** (A) Schematic representation of the *CG97175* locus in the left arm of the second chromosome of the *Drosophila melanogaster* genome. Orange boxes denote the coding sequence. The location of the WD40 repeat and transmembrane (TM) domains are shown on a representation of the spliced mRNA below. Blue boxes mark the locations within the *CG9175* mRNA of the target sequences of the two interfering shRNAs used in this study. (B) Domain organization in orthologous Sec12 proteins from *Drosophila melanogaster* (RefSeq NP_608995.2), *Homo sapiens* (NP_037520.1) and *Saccharomyces cerevisiae* (NP_014423.1). Percentage identity and similarity relative to *Drosophila* Sec12 (CG9175) are shown. (C) Confocal image of the distribution of Sec12 (UAS-Sec12–CFP, green) and ER marker Sec61β (UAS-Sec61β–tdTom, magenta) expressed in adipocytes of wild-type fat body. Nuclei stained with DAPI (cyan). Images on the upper right (Sec12–CFP and Sec61β–tdTom) and bottom right (Sec12–CFP alone) show magnified views of the area highlighted by the dashed square. (D) Distribution of Collagen IV (endogenous Vkg–GFP, green) in wild-type fat body (left), where it is detected only in surrounding basement membrane (BM) and Collagen IV intercellular concentrations (CIVICs, circles), and fat body where Sec12 has been knocked down with two independent RNAi transgenes (center and right). Nuclei stained with DAPI (blue). (E) Distribution of general secretion marker secreted GFP (signal peptide of Wingless coupled to GFP, green) expressed in wild type fat body (left) and fat body where Sec12 has been knocked down with two independent RNAi transgenes (center and right). Nuclei stained with DAPI (blue). Images in C–E are representative of three experimental repeats.

### Loss of Sec12 uncouples Sec16 and Tango1

To investigate the function of *Sec12*, we searched for transgenic RNAi lines targeting this gene, but found none were available in the three major collections (Kyoto NIG, Vienna VDRC and Harvard TRiP). Therefore, we generated two independent transgenic lines to express, under control of the GAL4-UAS system, interfering shRNAs targeting non-overlapping regions of the *Sec12* mRNA. In contrast to what is seen in wild-type fat body, where Collagen IV is detected exclusively in the surrounding basement membrane and in intercellular CIVICs (Collagen IV intercellular concentrations; Dai et al., 2017), expression of either shRNA in the larval fat body led to intracellular retention of Collagen IV, visualized using endogenously tagged Vkg–GFP (Fig. 3D). This intracellularly retained Collagen IV localized to rounded structures positive for the ER marker KDEL–RFP (Fig. S3), indicating that retention took place inside dilated ER cisternae. Furthermore, secretion of general secretory reporter secr-GFP, consisting of a signal peptide fused to GFP, was similarly impaired upon Sec12 depletion (Fig. 3E), indicating that *Drosophila* Sec12 is required for general secretion. To further characterize the role of Sec12 in secretory pathway function, we examined the localization of Sec16 and Tango1 following Sec12 knockdown. In the wild type, Sec16 and Tango1, visualized using Sec16–sGFP and anti-Tango1 immunostaining, tightly colocalized at ERES. However, upon Sec12 knockdown, their association was markedly reduced and frequently appeared as separated puncta (Fig. 4A; quantified in Fig. 4B). Quantification using Pearson's correlation and Manders' overlap coefficients confirmed reduced Tango1–Sec16 colocalization, consistent with their dissociation upon Sec12 knockdown (Fig. 4C). Furthermore, the size of puncta containing only Tango1 or only Sec16 was significantly reduced compared with that in the wild type, whereas the size of puncta retaining both proteins was not significantly affected (Fig. 4D). Finally, quantification of puncta abundance following Sec12 depletion revealed no significant difference in the total number of puncta relative to that in the wild type when puncta containing only Tango1, only Sec16, or both proteins were considered together (Fig. 4E). Thus, our data indicate that Sec12 plays a crucial function in secretory pathway architecture, promoting the coupled presence of Sec16 and Tango1 at ERES.

**Fig. 4.**
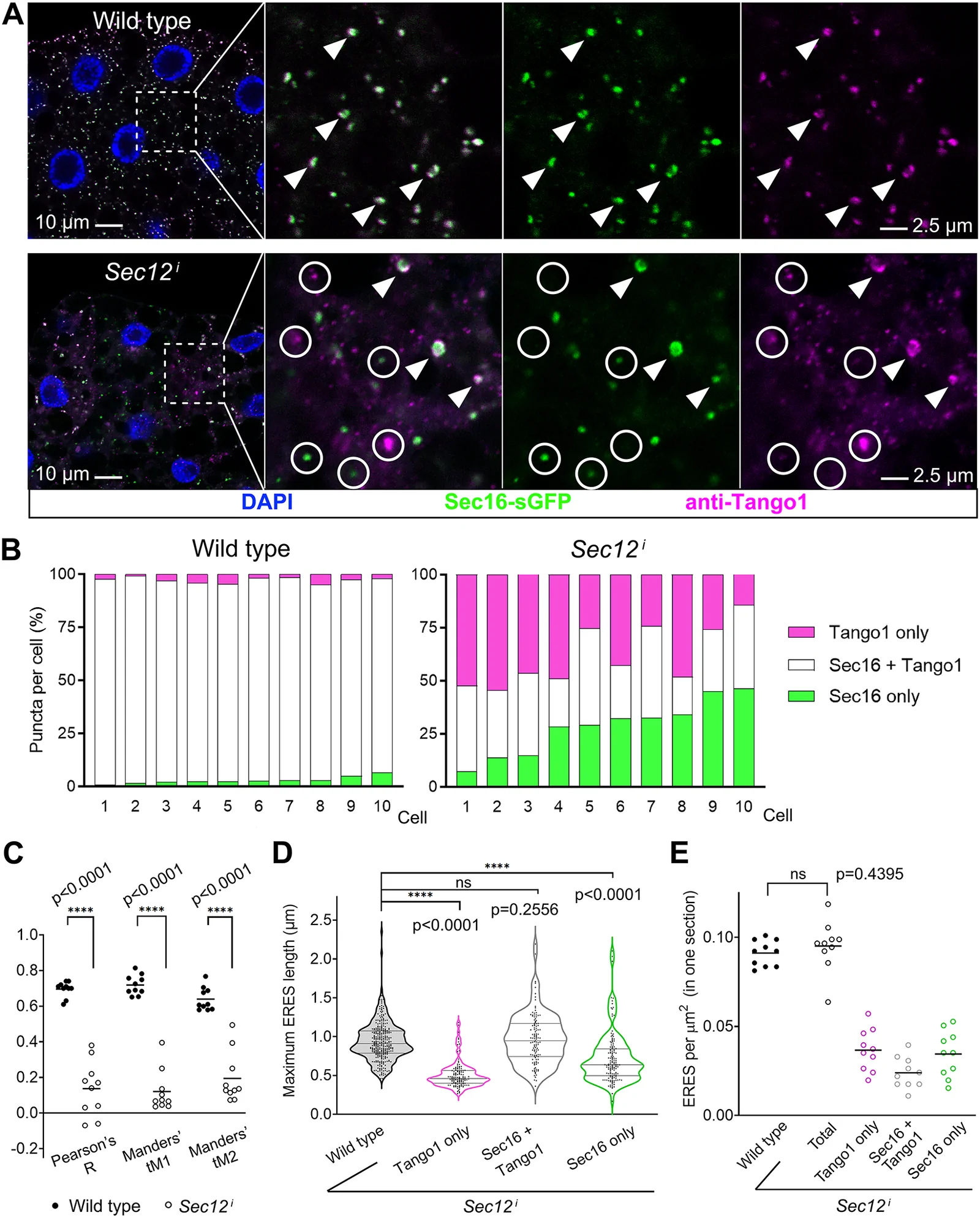
**Loss of Sec12 uncouples Sec16 and Tango1.** (A) Distribution of Sec16 (Sec16–sGFP, green) and Tango1 (anti-Tango1 antibody, magenta) in wild-type (top row panels) and *Sec12^i^* (bottom row panels) fat body. Nuclei stained with DAPI (blue). The area highlighted by the dashed square is magnified in center left, center right and right panels, showing Tango1 (right), Sec16 (center right) and both combined (center left). Arrowheads point to puncta positive for both Sec16 and Tango1; circles mark isolated Sec16 or Tango1 puncta. (B) Graphs quantifying the association of Tango1 and Sec16 in wild-type (left graph) and *Sec12^i^* (right graph) individual fat body adipocytes. For each cell analyzed by counting all puncta, the percentage of puncta where Tango1 and Sec16 overlap is shown in white, whereas the percentage of single Sec16 and single Tango1 puncta are shown in green and magenta, respectively. *n*=10 cells per genotype and ≥29 puncta per cell. (C) Graph quantifying Tango1–Sec16 colocalization through Pearson's and Manders' coefficients after Costes thresholding in wild-type and *Sec12^i^* individual fat body adipocytes. The mean is highlighted; *n*=10 cells per genotype. (D) Graph comparing ERES size in wild-type and *Sec12^i^* fat body adipocytes. Each dot represents a measurement of the maximum length of a punctum. Violin plots are shown with median value and interquartile range marked; *n*≥119. (E) Graph comparing ERES abundance in wild-type and *Sec12^i^* fat body adipocytes. The mean is highlighted; each dot represents a measurement of the density of puncta counted inside a square of the dimensions of the insets in A and E, *n*=10. ****P<0.0001; ns, not significant for means compared with wild type (two-tailed unpaired *t*-tests).

### *Drosophila* Tango1 interacts with Sec12 and Sec16

We next examined whether Sec16 and Tango1 influence the localization of Sec12. When Sec12–CFP was expressed in the larval fat body under the control of Cg-GAL4, it displayed a homogeneous ER distribution. In contrast, Tango1 overexpression induced redistribution of Sec12, promoting its concentration into large puncta and circular structures, whereas Sec16 overexpression also promoted Sec12 concentration to a lesser extent (Fig. 5A). Upon Tango1 overexpression, the ER marker Sec61β-tdTom retained its homogeneous reticular distribution, unlike Sec12 (Fig. 5B). This result shows that the redistribution of Sec12 induced by Tango1 was specific and did not reflect a general ER reorganization. Moreover, co-overexpression of Tango1 and Sec16 led to the formation of even larger structures in which Sec12 was prominently concentrated (Fig. 5C). These observations indicate that despite Sec12 general ER distribution, Tango1 and, to a lesser extent Sec16, can recruit or stabilize Sec12 at specialized ER subdomains, consistent with a coordinated role for all three proteins in ERES definition. Supporting this model, co-immunoprecipitation experiments using an antibody against *Drosophila* Tango1 demonstrated that Tango1 associates with both FLAG-tagged Sec16 (Fig. 5D; Fig. S4) and CFP-tagged Sec12 (Fig. 5E; Fig. S4). These biochemical interactions are consistent with direct or complex-mediated protein–protein interactions and provide support for their mutual regulation at ERES.

**Fig. 5.**
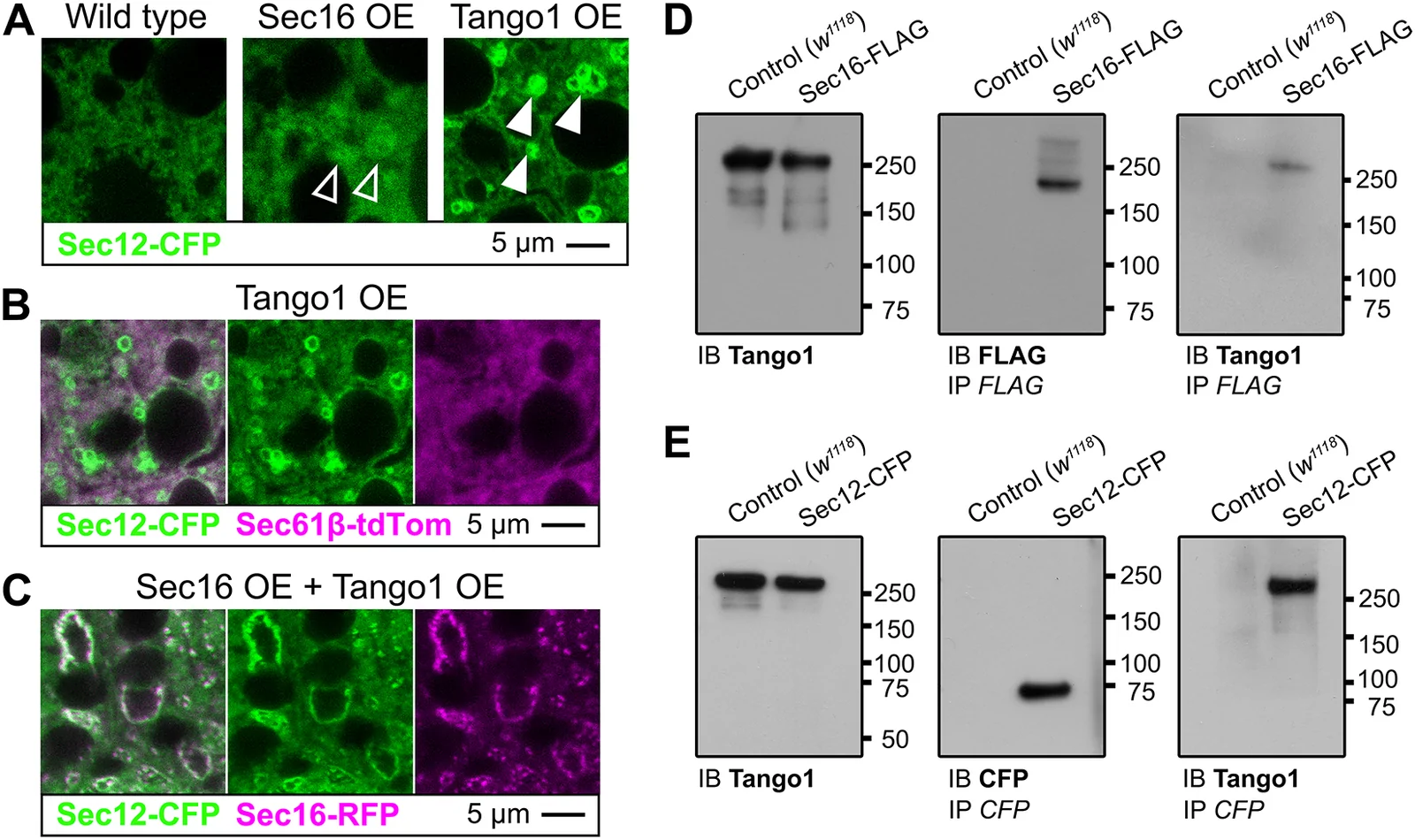
***Drosophila* Tango1 interacts with Sec12 and Sec16.** (A) Sec12 distribution (UAS-Sec12–CFP, green) in adipocytes of wild-type (left), Sec16-overexpressing (center) and Tango1-overexpressing (right) fat body. Arrowheads point to Sec12 concentrations. (B) Distribution of Sec12 (UAS-Sec12–CFP, green) and ER marker Sec61 (UAS-Sec61β–tdTom, magenta) in Tango1-overexpressing fat body. Images show Sec12 and Sec61 signals together (left panel) and separately (center and right panels). (C) Distribution of Sec12 (UAS-Sec12–CFP, green) in fat body co-overexpressing Sec16 (UAS-Sec16–RFP, magenta) and Tango1 (untagged). Images show Sec12 and Sec16 signals together (left panel) and separately (center and right panels). (D) Fat body lysates from wild-type control flies (*w^1118^*) and flies expressing Sec16–FLAG under control of endogenous promoter were immunoblotted (IB) with anti-Tango1 antibody (left panel), immunoprecipitated (IP) and immunoblotted with anti-FLAG antibody (center panel), and finally immunoblotted with anti-Tango1 after immunoprecipitation with anti-FLAG (right panel) to show Sec16–Tango1 co-immunoprecipitation. (E) Fat body lysates from wild type control flies (*w^1118^*) and flies expressing Sec12–CFP under control of Cg-GAL4 were immunoblotted (IB) with anti-Tango1 antibody (left panel), immunoprecipitated (IP) and immunoblotted with anti-CFP antibody (center panel), and finally immunoblotted with anti-Tango1 after immunoprecipitation with anti-CFP (right panel) to show Sec12–Tango1 co-immunoprecipitation. Images are representative of at least three experimental repeats.

### ERES–Golgi coupling depends on Tango1, but not Sec16

To further investigate the respective roles of Sec16 and Tango1, we examined the status of the Golgi within fat body ERES–Golgi units. To assess this, we performed immunostaining with an antibody against Golgin245, a marker of the trans-Golgi (Riedel et al., 2016). Consistent with our previous findings that *Drosophila* Tango1 mediates tethering between ERES and Golgi (Liu et al., 2017), Tango1 knockdown frequently resulted in Golgin245-positive puncta that were spatially separated from remaining Sec16–sGFP puncta (Fig. 6A; quantified in Fig. 6C), indicating a disruption of ERES–Golgi coupling. In contrast, Sec16 knockdown produced Tango1 puncta that were still associated in most cases with Golgin245-positive puncta (Fig. 6B; quantified in Fig. 6D), showing that physical proximity between Tango1-positive ERES and Golgi elements was preserved in the absence of Sec16. Furthermore, these differential effects of Tango1 and Sec16 were further supported by analyzing the relationship between ERES components and ER-retained cargo. Upon Tango1 depletion, Sec16 puncta appeared dissociated from ER-retained accumulations of Collagen IV (Fig. 6E). Conversely, upon Sec16 knockdown, Tango1 puncta remained associated with Collagen IV accumulations (Fig. 6F). Together, these findings reveal distinct functional contributions of Tango1 and Sec16 – although both are required for proper ERES formation, Tango1 plays a prominent role in maintaining ERES–Golgi coupling, as revealed by their respective loss of function phenotypes.

**Fig. 6.**
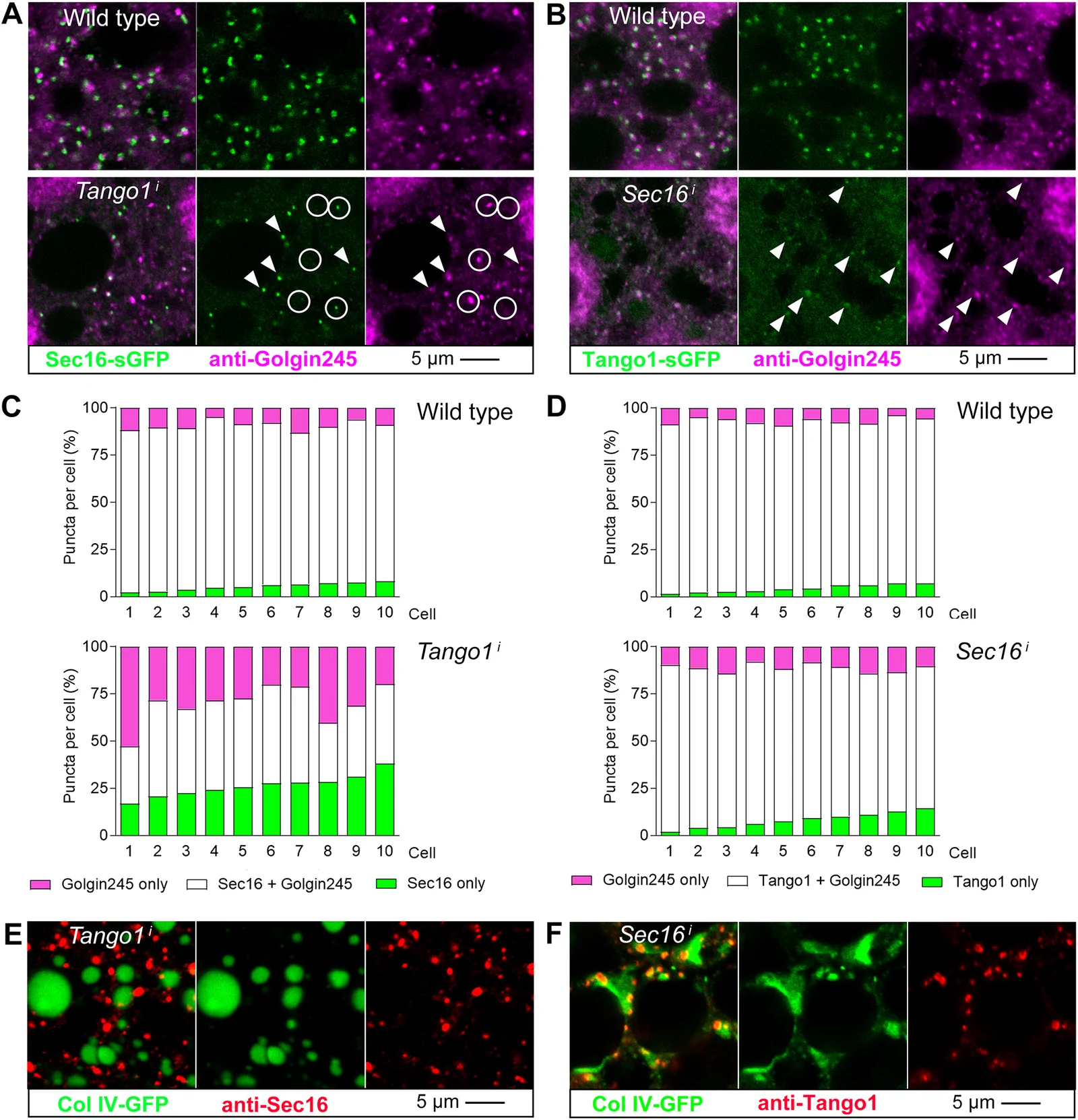
**ERES-Golgi coupling depends on Tango1, but not Sec16.** (A) Distribution of Sec16 (Sec16–sGFP, green) and Golgi (anti-Golgin245 antibody, magenta) in wild-type (top row panels) and *Tango1^i^* (bottom row panels) fat body. Images show Sec16 and Golgin245 signals together (left panel) and separately (center and right panels). Arrowheads point to puncta positive for both Sec16 and Golgin245; circles mark isolated Sec16 or Golgin245 puncta. (B) Distribution of Tango1 (Tango1–sGFP, green) and Golgi (anti-Golgin245 antibody, magenta) in wild-type (top row panels) and *Sec16^i^* (bottom row panels) fat body. Images show Tango1 and Golgin245 signals together (left panel) and separately (center and right panels). Arrowheads point to puncta positive for both Tango1 and Golgin245; there are no isolated Tango1 or Golgin245 puncta. (C) Graphs quantifying the association of Sec16 and Golgin245 in wild-type (top graph) and *Tango1^i^* (bottom graph) individual fat body adipocytes. For each cell analyzed by counting all puncta, the percentage of puncta where Tango1 and Sec16 overlap is shown in white, whereas the percentages of isolated Sec16 or Golgin245 puncta are shown in green and magenta, respectively. *n*=10 cells per genotype and ≥22 puncta per cell. (D) Graphs quantifying the association of Tango1 and Golgin245 in wild-type (top graph) and *Sec16^i^* (bottom graph) individual fat body adipocytes. For each cell analyzed by counting all puncta, the percentage of puncta where Tango1 and Golgin245 overlap is shown in white, whereas the percentages of isolated Tango1 or Golgin245 puncta are shown in green and magenta, respectively. *n*=10 cells per genotype and ≥19 puncta per cell. (E) Distribution of Sec16 (anti-Sec16, red) and ER-retained Collagen IV (Vkg-GFP, green) in *Tango1^i^* fat body. Images show Sec16 and Col IV signals together (left panel) and separately (center and right panels). Notice Sec16 puncta are separated from Col IV accumulations. (E) Distribution of Tango1 (anti-Tango1, red) and ER-retained Collagen IV (Vkg-GFP, green) in *Sec16^i^* fat body. Images show Tango1 and Col IV signals together (left panel) and separately (center and right panels). Notice Tango1 puncta associate with Col IV accumulations. Images in E and F are representative of three experimental repeats.

### Effects of Tango1 and Sec16 loss on recruitment of COPII and COPI to ERES

To additionally assess the roles of Sec16 and Tango1 in establishing a functional ERES, we examined their impact on the recruitment of COPII and COPI coat proteins, which mediate anterograde and retrograde trafficking at the ER–Golgi interface, respectively. To do this, we simultaneously imaged the distribution of COPII component Sec13–GFP and the COPI subunit γCOP–RFP, both expressed under control of Cg-Gal4, in fat body stained with anti-Sec16 or anti-Tango1 antibodies. Knockdown of Tango1 led to a marked reduction in the concentration of both Sec13 and γCOP, in contrast to what was seen in the wild type, where both proteins form distinct puncta, Tango1-depleted cells lacked clearly discernible COPII and COPI puncta (Fig. 7A). To quantify this effect, we measured the mean Sec13 and γCOP fluorescence associated with Sec16-positive puncta in control and Tango1 RNAi conditions. Upon Tango1 depletion, only 32% of the Sec13 signal and 6% of the γCOP signal remained relative to wild-type levels (Fig. 7B). These data indicate a strong requirement for Tango1 in the concentration of both COPII and COPI components at ERES. Sec16 knockdown produced a partially distinct phenotype. Similar to Tango1 depletion, γCOP concentration was almost completely lost. However, Sec13-positive puncta were still detectable, albeit reduced in size and associated with smaller Tango1 puncta (Fig. 7C; Fig. S5A,B). Quantification revealed that 79% of the Sec13 signal remained associated with Tango1-positive structures, whereas only 4% of the γCOP signal persisted compared to wild-type levels (Fig. 7D). Thus, Sec16 is absolutely required for COPI concentration while also significantly contributing to COPII retention at ERES. In summary, both Tango1 and Sec16 are necessary for efficient recruitment of COPII and COPI coat proteins to ERES, albeit with distinct relative contributions. Together with our findings on Sec12, these results support a model in which Sec12, Sec16 and Tango1 cooperate to define and maintain functional ERES architecture and to ensure proper bidirectional trafficking at the ER–Golgi interface (Fig. 7E,F).

**Fig. 7.**
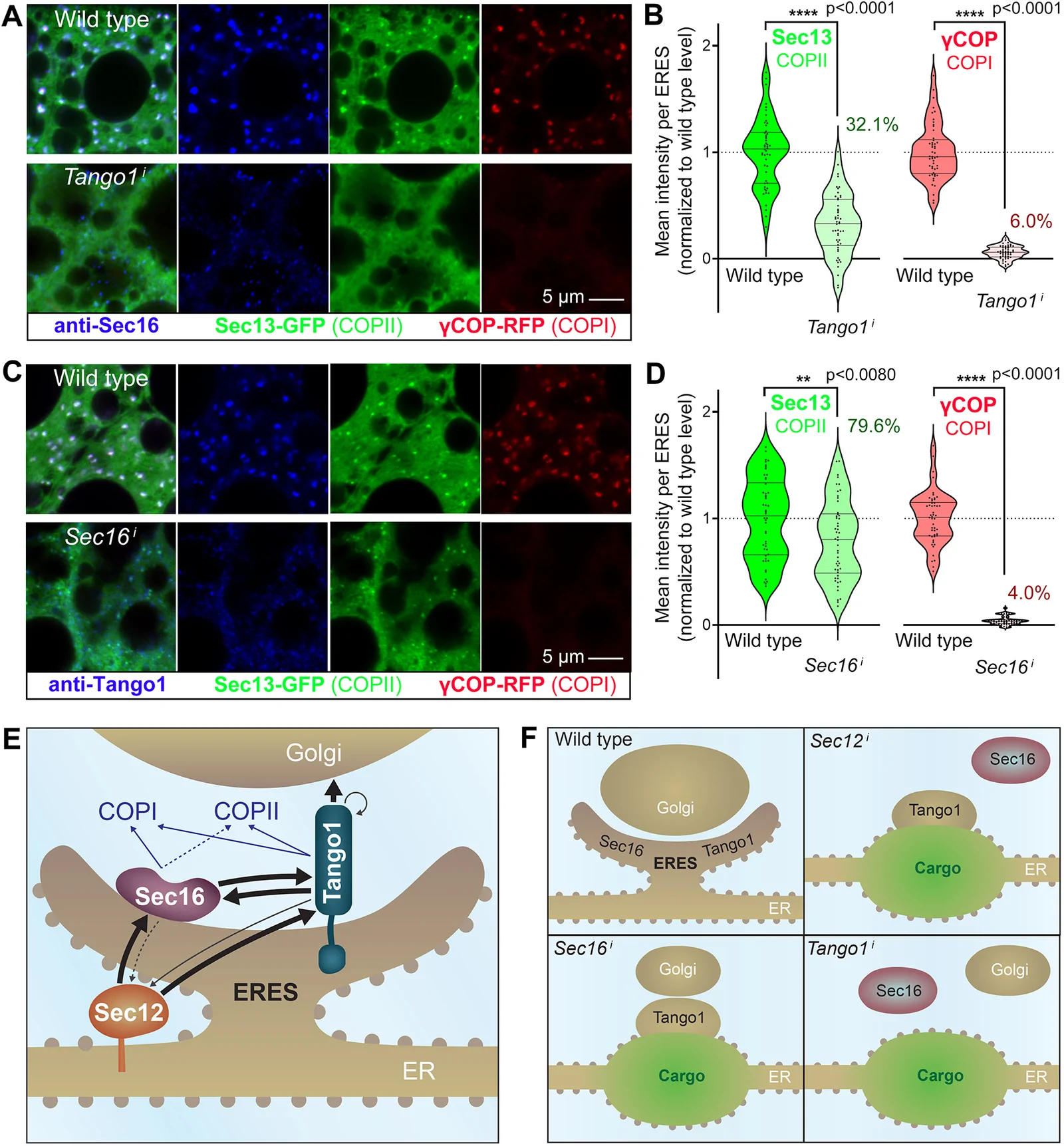
**Effects of Tango1 and Sec16 loss on recruitment of COPII and COPI to ERES.** (A) Distribution of Sec16 (anti-Sec16, blue), Sec13 (UAS-Sec13–GFP, green) and γCOP (UAS-γCOP–RFP, red) in wild-type (top row panels) and *Tango1^i^* (bottom row panels) fat body. Images show Sec16, Sec13 and γCOP together (left panel) and separately (center left, center right and right panels). (B) Graphs quantifying the intensity of Sec13 (left) and γCOP (right) signals associated with Sec16 puncta in wild-type and *Tango1^i^* fat body. Violin plots are shown with median value and interquartile range marked; *n*=50. Each dot represents a measurement of mean intensity in a Sec16-associated Sec13 or γCOP punctum. Compared to the wild-type, mean values in *Tango1^i^* (32.1% of the wild type for Sec13 and 6.0% for γCOP) were significantly different. (C) Distribution of Tango1 (anti-Tango1, blue), Sec13 (UAS-Sec13-GFP, green) and γCOP (UAS-γCOP–RFP, red) in wild type (top row panels) and *Sec16^i^* (bottom row panels) fat body. Images show Tango1, Sec13 and γCOP together (left panel) and separately (center left, center right and right panels). (D) Graphs quantifying the intensity of Sec13 (left) and γCOP (right) signals associated with Tango1 puncta in wild-type and *Sec16^i^* fat body. Violin plots are shown with median value and interquartile range marked; *n*=50. Each dot represents a measurement of mean intensity in a Tango1-associated Sec13 or γCOP punctum. Compared to the wild type, mean values in *Sec16^i^* (79.6% of the wild type for Sec13 and 4.0% for γCOP) were significantly different. ****P<0.0001; **P<0.01 for means compared with wild type (two-tailed unpaired *t*-tests). (E) Model depicting the interactions among Tango1, Sec16 and Sec12 and their roles in defining functional ERES. ER-localized Sec12 initiates ERES assembly, bringing together Sec16 and Tango1, which in turn stabilize each other's concentration at specific ER domains. Apart from their mutual interaction, Sec16 and Tango1 have roles in maintaining efficient traffic at the ER–Golgi interface through tethering of Golgi membranes and self-interaction (Tango1), COPII concentration (Tango1 and, to a lesser extent, Sec16) and COPI concentration (both Sec16 and Tango1). (F) Schematic representation of the effects of Sec12, Sec16 and Tango1 loss on the organization of the ER–Golgi interface.

## DISCUSSION

The organization of ERES is central to the fidelity and efficiency of the secretory pathway. Here, we used *Drosophila melanogaster* to dissect the respective roles of Sec16, Tango1 and Sec12 in ERES morphogenesis and function, taking advantage of the fact that single homologs exist for each protein in this organism. Using transgenic tools that enable localization and functional analysis in a physiological *in vivo* context, we conducted our study in the larval fat body, a tissue responsible for secreting most hemolymph (insect blood) proteins, including extracellular matrix components, clotting factors and circulating lipoproteins. Altogether, our results support a model in which Sec16, Tango1 and Sec12 act interdependently to define functional ERES while making distinct contributions to ERES architecture, ER–Golgi coupling and coat recruitment.

Loss- and gain-of-function analyses reveal a strong reciprocal dependence between Sec16 and Tango1. Depletion of either protein reduces both the size and abundance of ERES. Because Tango1 is an integral membrane protein, residual Tango1 concentrations observed upon Sec16 knockdown are likely to continue marking specific ER subdomains. In contrast, the nature of Sec16 accumulations detected after Tango1 depletion is less clear. As a peripheral membrane protein, Sec16 could remain associated with ER membranes or redistribute into membraneless assemblies resembling Sec bodies described under stress conditions (Zacharogianni et al., 2014). Conversely, overexpression of either Sec16 or Tango1 enlarges ERES and promotes recruitment of the other protein, with co-overexpression producing synergistically enlarged structures. Overall, these symmetrical phenotypes strongly argue against a linear hierarchical relation. Instead, they favor a scenario in which Sec16 and Tango1 mutually stabilize or scaffold one another at ERES. Although Sec16 has long been recognized as a COPII scaffold and organizer of ER export, Tango1 was initially proposed to function as a collagen receptor in mammalian cells (Saito et al., 2009). However, our findings here, together with previous ones (Feng et al., 2021; Liu et al., 2017), support the view that Tango1 acts instead as a core structural component of ERES required for general secretion.

In addition to studying Sec16 and Tango1, our study identified the product of gene *CG9175* as the *Drosophila* ortholog of Sec12 and provides its first functional characterization in this organism. We show that Sec12 is required for efficient secretion, maintaining the coordinated localization of Sec16 and Tango1. Upon Sec12 depletion, these two proteins lose their tight colocalization and frequently segregate into distinct puncta, suggesting that Sec12 acts upstream in establishing ERES organization. Consistent with this idea, recent work in *Saccharomyces cerevisiae* has shown that Sec16 localization to ERES depends on the Sec12 paralog Sed4 (Yorimitsu and Sato, 2023), although Sec12 itself had previously been considered dispensable (Okamoto et al., 2012). Despite this upstream role of Sec12, the ability of overexpressed Tango1, and to a lesser extent of overexpressed Sec16, to recruit *Drosophila* Sec12 from the general ER to ERES suggests that once assembled these components operate within a mutually reinforcing network. The co-immunoprecipitation of *Drosophila* Tango1 with both Sec16 and Sec12 further supports the existence of such a cooperative regulatory module.

Despite their cooperative behavior downstream of Sec12, Sec16 and Tango1 make distinct functional contributions. Tango1 depletion disrupts spatial coupling between ERES and Golgi elements, leading to frequent dissociation of Golgi markers from residual Sec16 puncta. In contrast, although Sec16 knockdown reduces ERES size, Tango1-positive puncta remain associated with Golgi structures. These findings align with previous reports that Tango1 contributes to tethering ERES to Golgi or post-ERES membranes in both *Drosophila* (Liu et al., 2017) and mammalian cells (Nogueira et al., 2014). In addition to maintaining proximity with Golgi, functional ERES must coordinate bidirectional trafficking at that ER–Golgi interface, integrating COPII-mediated anterograde export with COPI-dependent retrograde recycling. Our analyses show that both Sec16 and Tango1 are required for the concentration of COPII and COPI components at ERES, albeit with different relative impacts. Tango1 depletion markedly reduces both COPII and COPI signals, whereas Sec16 depletion strongly impairs COPI recruitment but retains a substantial fraction of COPII signal associated with residual Tango1 puncta. Given that Sec16 is widely recognized as a scaffold for COPII assembly, the relatively moderate reduction in COPII enrichment observed here after Sec16 depletion is somewhat unexpected. One possibility is that, compared to *S. cerevisiae*, in animals, Tango1 assumes a large portion of this COPII-concentrating role, as its proline-rich domain can bind COPII components in both mammalian cells (Saito et al., 2009) and *Drosophila* (Liu et al., 2017). In contrast to the interactions between ERES proteins and the COPII machinery, which have been characterized and are considered central to ERES assembly and function, possible mechanisms by which Tango1 and Sec16 recruit COPI to ERES remain unexplored. Given the close association of COPI with ERES, where COPI localizes to the periphery of *Drosophila* ERES cups (Yang et al., 2021), it is tempting to speculate that direct physical interactions between COPI components and these ERES proteins contribute to ERES organization. Defining these interactions will be an important objective for future studies and might provide new insight into how anterograde and retrograde trafficking are coordinated at the ER–Golgi interface.

Beyond COPII, Sec16 and Sec12, confirmed Tango1 interactions include multiple conserved, more-ancient components of the early secretory pathway, such as Rab1, GRASP65 (Liu et al., 2017), p24 proteins (Yang et al., 2024) and the Syntaxin-18-interacting protein SLY1 (Nogueira et al., 2014). Thus, although Tango1 emerged later in evolution and is restricted to metazoans, it has become deeply integrated into the conserved molecular network that defines ERES. Interestingly, Sec16 in the round worm *Caenorhabditis elegans* and mammals interacts with TFG-1, a protein absent in flies but with a comparable role in tethering ERES to post-ERES membranes (Witte et al., 2011), illustrating the adaptability of the ERES organizational network across species. The evolutionary emergence of Tango1 in metazoans and its integration into ERES regulatory mechanisms might have contributed additional stability or regulatory complexity to ER export, a feature that could have been further elaborated by the diversification of Tango1 family proteins in certain lineages, such as mammals.

## MATERIALS AND METHODS

### *Drosophila* strains

Standard fly husbandry techniques and genetic methodologies, including balancers and dominant genetic markers, were used to assess segregation of mutations and transgenes in the progeny of crosses, construct intermediate fly lines and obtain flies of the required genotypes for each experiment (Roote and Prokop, 2013). Cultures were maintained at 25°C in all experiments. The GAL4-UAS binary expression system (Brand and Perrimon, 1993) was used to drive expression of UAS transgenes under temporal and spatial control of transgenic GAL4 drivers Cg-GAL4 and BM-40-SPARC-GAL4 for fat body, and rn-GAL4 for the wing disc. Genotypes of animals in all experiments are detailed in Table S1. The following strains were used: *w^1118^* (X) (BDSC 3605), *w ; Cg-GAL4* (II) (BDSC 7011), *w ; BM-40-SPARC-GAL4 / TM6B* (BDSC 77473), *y w ; Tango1-sGFP^fTRG00602^* (III) (VDRC v318173), *y w ; Sec16-sGFP^fTRG01259^* (III) (VDRC v318329), *w ; UAS-RFP-Sec16.22-2* (II) (this study), *w ; UAS-RFP-Sec16.14-2* (III) (this study), *w ; UAS-Tango1-FLAG.8B* (II) (VDRC v311426), *w ; UAS-SP-GFP-Tango1.20B* (III) (VDRC v311432), *y v ; UAS-Sec16.RNAi^TRiP.HMJ21252^* (II) (BDSC 53917), *w ; UAS-Tango1.RNAi^NIG.11098R-3^* (III) (NIG 10098R), *w ; UAS-Sec12-CFP.8* (III) (this study), *y sc v ; UAS-Sec12.RNAi^FZ1^* (II) (this study), *y sc v ; UAS-Sec12.RNAi^FZ2^* (II) (this study), *w ; UAS-tdTomato-Sec61β* (III) (BDSC 64747), *y w ; vkg^G454^-GFP/ CyO* (BDSC 98343), *w ; UAS-secr-GFP* (II) (gift from Jean-Paul Vincent, The Francis Crick Institute, London, UK), *w ; UAS-RFP-KDEL / TM3, Sb* (BDSC 30909), *w ; rn-GAL4 / TM3, Sb Ser* (BDSC 7405), *w ; UAS-Sec13-EGFP-APEX2.18-1* (III) (VDRC v311475), *y w ; If / CyO ; UAS-γCOP-mRFP* (BDSC 29714), *w ; UAS-EGFP-APEX2-Sec24CD(PC).30-2* (III) (VDRC v311491), *y w ; Sar1^CA07674^-GFP / TM3, Ser Sb* (BDSC 51180), *w ; UAS-γCOP-APEX2-EGFP.3.1* (III) (VDRC v311477), *y w ; αCOP-sGFP^fTRG01193^* (III) (VDRC v318793), *w ; UAS-Arf1-EGFP-APEX2.16.3* (III) (VDRC v311479).

### Generation of transgenic lines

#### UAS-RFP-Sec16

The coding sequence of Sec16 was amplified by PCR from whole L3 larva cDNA synthesized using PrimeScript RT-PCR Kit (Takara, cat. no.RR014-A). Primers used were: attSec16-F, 5′-GGGGACAAGTTTGTACAAAAAAGCAGGCTTCATGTTGCACAATAATGCGCCCT-3′; attSec16-R, 5′-GGGGACCACTTTGTACAAGAAAGCTGGGTCCTACTGCTGGGGTACAAAGT-3′.

The fragment obtained was purified by gel extraction using AxyPrep DNA Gel Extraction Kit (Axygen, cat. no. AP-GX-250G) and cloned into vector pDONR221 (Life Technologies, cat. no. 12536017) with Gateway BP Clonase Enzyme Mix (Life Technologies, cat. no. 11789020) to obtain entry clone pDONR221-Sec16. From this entry clone, the Sec16 sequence was transferred into destination vector pTRW (UASt, N-terminal mRFP, *Drosophila* Carnegie Vector collection) using Gateway LR Clonase Enzyme Mix (Life Technologies, cat. no. 12538120).

#### UAS-Sec12-CFP

The coding sequence of Sec12 was amplified by PCR from whole L3 larva cDNA synthesized using PrimeScript RT-PCR Kit (Takara, cat. no.RR014-A). Primers used were: attSec12-F, 5′-GGGGACAAGTTTGTACAAAAAAGCAGGCTTCATGGCCCACACCCGCCGTC-3′; attSec12-R, 5′-GGGGACCACTTTGTACAAGAAAGCTGGGTCGATGCCAATGTAGGAGCACA-3′.

The fragment obtained was purified by gel extraction using AxyPrep DNA Gel Extraction Kit (Axygen, cat. no. AP-GX-250G) and cloned into vector pDONR221 (Life Technologies, cat. no. 12536017) with Gateway BP Clonase Enzyme Mix (Life Technologies, cat. no. 11789020) to obtain entry clone pDONR221-Sec12. From this entry clone, the Sec12 sequence was transferred into destination vector pTWC (UASt, C-terminal ECFP, *Drosophila* Carnegie Vector collection) using Gateway LR Clonase Enzyme Mix (Life Technologies, cat. no. 12538120).

#### UAS-Sec12.RNAi

Two short hairpin oligonucleotides to knockdown expression of Sec12 were designed using DSIR (https://biodev.cea.fr/DSIR/DSIR.html): Sec12-sh-F1, 5′-CTAGCAGTCAGTCCCGACAGCAAGCTAATTAGTTATATTCAAGCATAATTAGCTTGCTGTCGGGACTGGCG-3′; Sec12-sh-R1, 5′-AATTCGCCAGTCCCGACAGCAAGCTAATTATGCTTGAATATAACTAATTAGCTTGCTGTCGGGACTGACTG-3′; Sec12-sh-F2: 5′-CTAGCAGTCGCACTGTCAGATGTACTACGTAGTTATATTCAAGCATACGTAGTACATCTGACAGTGCGGCG-3′; Sec12-sh-R2, 5′-AATTCGCCGCACTGTCAGATGTACTACGTATGCTTGAATATAACTACGTAGTACATCTGACAGTGCGACTG-3′.

After annealing complementary oligonucleotides (F1 with R1 and F2 with R2), the products were inserted into the VALIUM22 vector, which had been previously linearized by NheI and EcoR1 double restriction (New England Biolabs, cat. no. R3131 and R3101, respectively). The resulting plasmids were transformed, miniprepped with QIAprep Spin Miniprep kit (QIAGEN, cat. no. 27104) and injected into fly strain *y v sc nos-integrase ; attP40* for stable transgene integration. Transgenic lines generated in this study will be made available through the VDRC and/or BDSC stock centers.

### Imaging and image analysis

For preparation of fat body or wing discs, larvae were pre-dissected in PBS by turning them inside-out with fine-tip forceps, fixed in PBS containing 4% paraformaldehyde (cat. no. 80096692; Sinopharm Chemical Reagent; 20 min), washed in PBS (3×10 min), and further processed for antibody and/or phalloidin staining (see the ‘Immunohistochemistry’ section) or finally dissected and mounted on a microscope slide with a drop of DAPI-Vectashield (Vector Labs, cat. no. H-1200). Confocal microscopy images were acquired in a Zeiss LSM780 microscope equipped with Plan-Apochromat objectives 63× oil (NA 1.4) and 100× oil (NA 1.4), or an Olympus FV1200 microscope equipped with a 60× oil (NA 1.4) objective. For ERES number measurements (Figs 1G, 2G,I), images were analyzed with the ‘Multi-point’ tool in Fiji-ImageJ. For ERES size measurements (Figs 1H, 2H,J), images were analyzed with the ‘Line’ tool in Fiji-ImageJ. For puncta association measurements (Figs 4B, 6C,D), images were analyzed with the ‘Multi-point’ tool in Fiji-ImageJ. Colocalization analysis (Fig. 4C) was performed with the Coloc2 plugin in Fiji-ImageJ, using Costes thresholding. For measurements of COPII and COPI ERES-associated signal (Fig. 7B,D), Tango1 and Sec16 puncta were delimited with the ‘Freehand selection’ tool in Fiji-ImageJ and the mean intensity of Sec13–GFP and γCOP–RFP were measured inside the selected area. Statistical analysis and graphical representations were performed with GraphPad Prism software. Statistical significance is reported in graphs as follows: ****P<0.0001; ***P<0.001; **P<0.01; *P<0.05; not significant (n.s.), *P*>0.05.

### Immunohistochemistry

The following primary antibodies were used: guinea pig anti-Tango1 (Lerner et al., 2013) (1:1000, RRID AB_2570287), rabbit anti-Sec16 (1:1000) (Ivan et al., 2008) and goat anti-Golgin245 (1:1000, RRID AB_2618260) (Riedel et al., 2016). Secondary antibodies were against IgG conjugated to Alexa-Fluor-488, Alexa-Fluor-555 or Alexa-Fluor-647, all used at 1:200 dilution (Life Technologies). Larvae were pre-dissected in PBS by turning them inside-out with fine-tip forceps, fixed in PBS containing 4% paraformaldehyde (cat. no. 80096692; Sinopharm Chemical Reagent; 20 min), washed in PBS (3×10 min), blocked in PBT-BSA [PBS containing 0.1% Triton X-100 detergent (cat. no. T8787; Sigma-Aldrich), 1% BSA (cat. no. 201903A28; Zhongkekeao), and 250 mM NaCl (cat. no. 0805C384; Amresco); 3×20 min], incubated overnight with primary antibody in PBT-BSA, washed in PBT-BSA (3×20 min), incubated for 2 h with secondary antibody in PBT-BSA, and washed in PBT-BSA (3×20 min) and, finally, in PBS (3×10 min). Phalloidin staining of wing discs were performed with Texas-Red–phalloidin (1:100 of stock solution in DMSO, Life Technologies, cat. no. T7471) by incubating samples with it for 2 h; this was done in PBT-BSA at the same time as secondary antibody incubation (Fig. S2D) or in PBT after 3×20 min PBT permeabilization washes when antibody staining was not being performed on the tissues (Fig. S2A–C). Fat body or wing discs were finally dissected and mounted on a slide with a drop of DAPI-Vectashield (Vector Labs, cat. no. H-1200).

### Immunoprecipitation

Larval fat body was dissected in PBS from 100–200 larvae and homogenized with a motorized pellet pestle in 200 μl ice-cold lysis buffer containing 10 Mm Tris-HCl (pH 7.5, Amresco, cat. no. 0497-500G), 150 mM NaCl (Amresco, cat. no. X 190), 0.5 mM EDTA (Xilonghuagong, cat. no. 60-00-4), 0.5% NP-40 (Loogene, cat. no.ZC01468) and protease inhibitor (Amresco, cat. no. M221-1ML). Lysates were cleared by centrifugation (20,000 ***g***, 30 min, 4°C) and the supernatant was transferred to a pre-cooled Eppendorf tube. Protein concentration was estimated by BCA assay (JK GREEN, cat. no. SD2006) with a Nanodrop 2000C spectrophotometer. To immunoprecipitate the desired proteins in this study, lysates were incubated with GFP-TrapA beads (Chromotek, cat. no. gta-20, also binding YFP) or anti-FLAG M2 magnetic beads (Sigma, cat. no. M8823-1ML) according to manufacturer's instructions. Proteins bound to beads were eluted before analysis by western blotting. For GFP beads, 50 μl of 2× SDS sample buffer (120 mM Tris-HCl pH 6.8, 20% glycerol, 4% SDS, 0.04% Bromophenol Blue, 10% β-mercaptoethanol) were added to beads, the mixture was incubated in a heat block at 95°C for 20 min and GFP beads were separated by centrifugation at 5000 ***g*** for 2 min at 4°C. For FLAG beads, 40 μl of a solution containing 48.5 μl of TBS buffer and 1.5 μl of 3× FLAG peptide stock solution [5 μg/μl of 3× FLAG peptide (ApexBio, cat. no. A6001), 100 mM Tris-HCl pH 7.5, 200 mM NaCl] were added to beads (150 ng/μl 3× FLAG final concentration), the mixture was incubated in a rotator for 1 h at 4°C and FLAG beads were collected with a magnetic separator and 10 μl of 5× SDS-PAGE loading buffer was added to samples (250 mM Tris-HCl pH 6.8, 50% glycerol, 10% SDS, 0.5% Bromophenol Blue, 5% β-mercaptoethanol).

### Western blotting

Fat body from 100 larvae was dissected in ice-cold PBS and homogenized with a motorized pellet pestle in 100 μl ice-cold lysis buffer containing 10 mM Tris-HCl (pH 7.5, Amresco, cat. no. 0497-500G), 150 mM NaCl (Amresco, cat. no. X 190), 0.5 mM EDTA (Xilonghuagong, cat. no. 60-00-4), 0.5% NP-40 (Loogene, cat. no. ZC01468), phosphatase inhibitor (Solarbio, cat. no. P1260 1:100) and protease inhibitor (Amresco, cat. no. M221-1ML, 1:100). Lysates were cleared by centrifugation (20,000 ***g*** for 30 min at 4°C) and supernatants transferred to pre-cooled Eppendorf tubes. Protein concentration was estimated by BCA assay (JK GREEN, cat. no. SD2006) with a Nanodrop 2000C spectrophotometer. Lysates or eluted immunoprecipitates were reduced by boiling 5 min at 95°C, separated (25 μl per lane) through SDS-PAGE in a 4–20% gradient gel (MiniProtean TGX, Bio-Rad, cat. no. 456-1093) and then electrophoretically transferred onto a nitrocellulose membrane (Bio-Rad, cat. no. 1620115) using a wet transfer system (Bio-Rad, cat. no. 1658034). The membranes were blocked with 10% milk in TBST (Oxoid, cat. no. LP0031), incubated with the primary antibody overnight, washed with TBST (4×10 min), incubated with HRP-coupled secondary antibody (1:20,000, 1 h), washed with TBST (4×10 min). Secondary antibodies were detected through autoradiography using ECL Plus chemiluminescence (Pierce, cat. no. 32132). Unprocessed blot images are shown in Fig. S4. Primary antibodies were anti-Tango1 (guinea pig, 1:5000, RRID AB_2570287), anti-FLAG (mouse, 1:2500, cat. no.RLM3001, Ruiying Biological), anti-GFP (recognizing also CFP, mouse, 1:5000, cat. no.AT0028, CMCTAG) and anti-β-actin (mouse, 1:2000, cat. no. AT0008, CMCTAG). The secondary antibodies were HRP-coupled anti-mouse (1:10,000, cat. no.M21001L, Abmart), anti-rabbit (1:10,000, cat. no. M21002L, Abmart), and anti-guinea pig (1:10,000, cat. no. ab6908, Abcam, RRID AB_955425). Precision Plus Protein Dual Color Standards (cat. no. 161-0394; Bio-Rad Laboratories) was used as a molecular mass marker.

## Supplementary Material



10.1242/joces.264950_sup1 Supplementary information
