# Supplementary material for: Cooperative definition of ER exit sites by Tango1, Sec16 and Sec12
Source: J Cell Sci. 2026 Sep 29;139(21):jcs264950. doi: 10.1242/jcs.264950 (PMC13723486; doi:10.1242/jcs.264950)
Supplement: Supplementary file 1 [file joces-139-264950-s1.pdf]

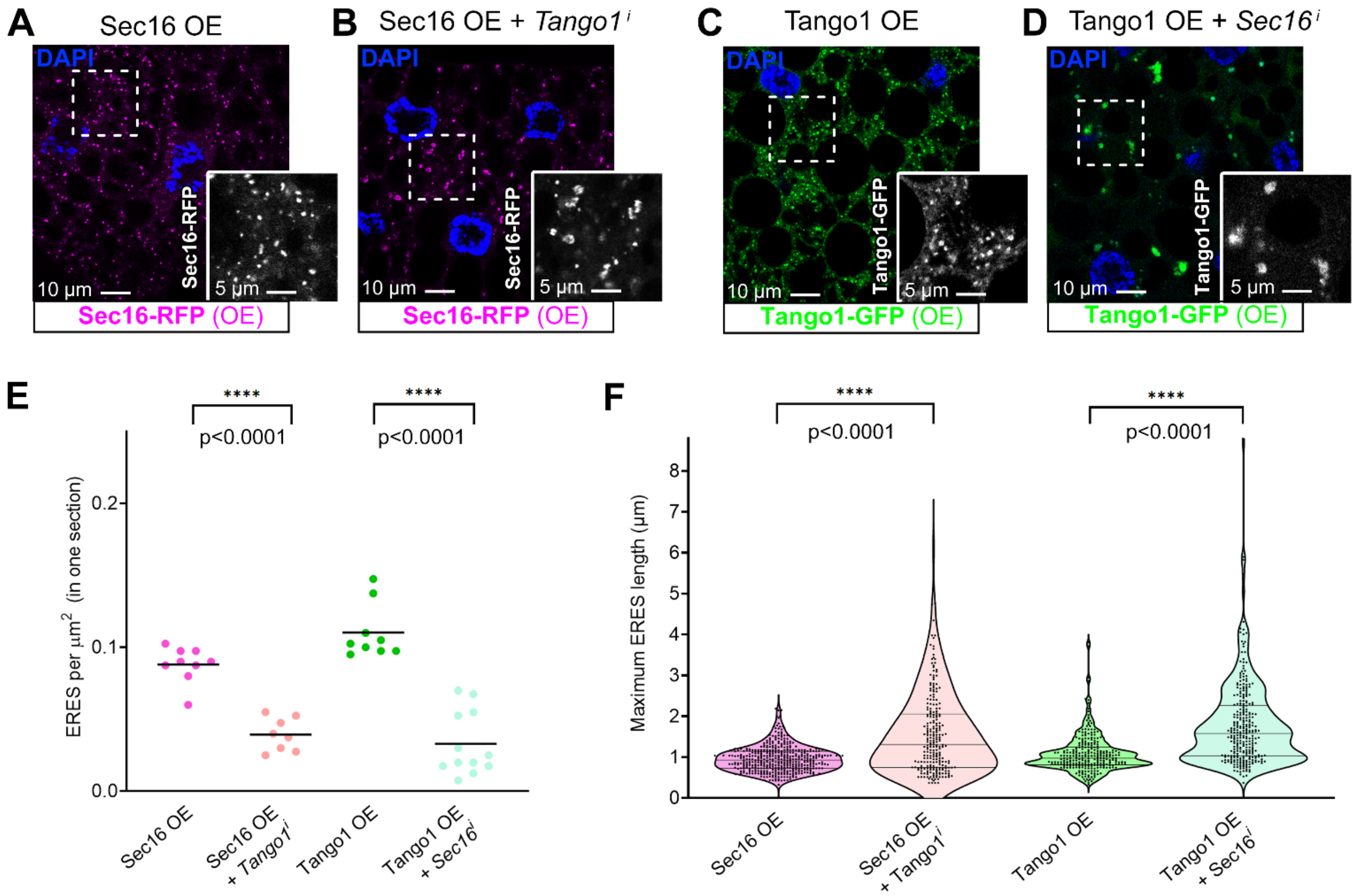

Fig. S1. Combined effects of Sec16 overexpression with Tango1 knock down and vice versa

(A) Distribution of overexpressed Sec16 (UAS-Sec16-RFP, magenta) in fat body adipocytes. Nuclei stained with DAPI (blue). Image in bottom right corner (Sec16-RFP, white) shows magnified view of inset (dashed square).

(B) Distribution of overexpressed Sec16 (UAS-Sec16-RFP, magenta) in fat body where Tango1 has been knocked down (Sec16 OE + *Tango1*^{i}). Image in bottom right corner (Sec16-RFP, white) shows magnified view of inset (dashed square).

(C) Distribution of overexpressed Tango1 (UAS-SP-GFP-Tango1, green) in fat body adipocytes. Nuclei stained with DAPI (blue). Image in bottom right corner (Tango1-GFP, white) shows magnified view of inset (dashed square).

(D) Distribution of overexpressed Tango1 (UAS-SP-GFP-Tango1, green) in fat body where Sec16 has been knocked down (Tango1 OE + *Sec16*^{i}). Image in bottom right corner (Tango1-GFP, white) shows magnified view of inset (dashed square).

(E) Graph comparing ERES abundance in Sec16 OE, Sec16 OE + *Tango1*^{i}, Tango1 OE and Tango1 OE + *Sec16*^{i} fat body adipocytes. Each dot represents a measurement of the density of puncta counted inside a square of the dimensions of the insets in A-D. n=10. Means were significantly different in two-tailed unpaired t tests, with p values reported in the graph.

(F) Graph comparing ERES size in Sec16 OE, Sec16 OE + *Tango1*^{i}, Tango1 OE and Tango1 OE + *Sec16*^{i}. fat body adipocytes. Each dot represents a measurement of the maximum length of a punctum. n≥245. Violin plots depict the median value and interquartile range. Significance of differences was determined through two-tailed unpaired t tests, with p values reported in the graph.

Journal of Cell Science • Supplementary information

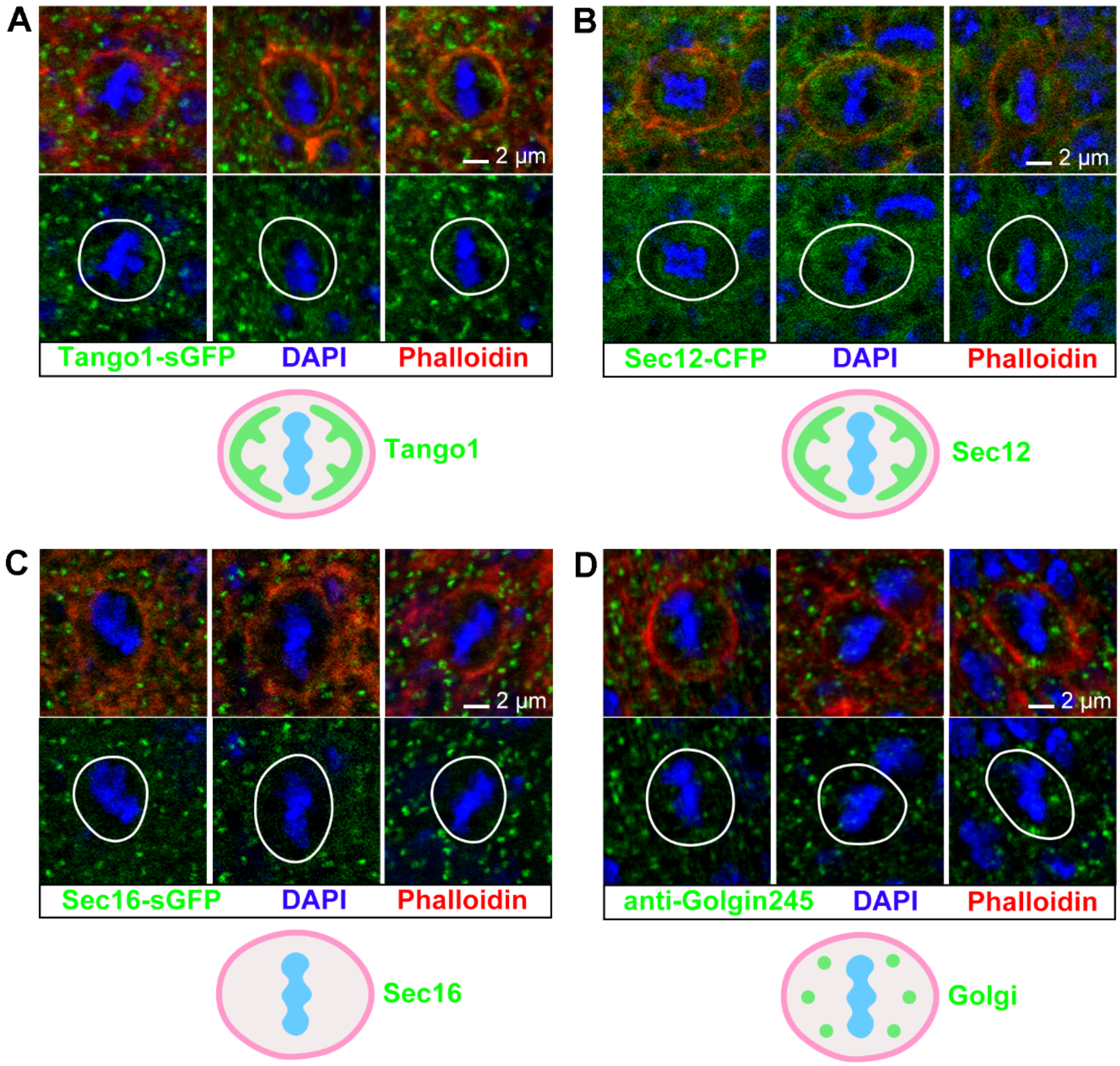

Fig. S2. Distribution of Tango1, Sec16 and Sec12 in dividing cells

(A) Distribution of Tango1 (Tango1-sGFP, green) in cells undergoing mitosis in the wing imaginal disc (prometaphase/metaphase stage). Phalloidin staining shown in red (F-actin). DNA stained with DAPI (blue). Top row panels show three examples, while bottom row panels show the same images with the dividing cells outlined in white and without the red (phalloidin) channel. The cartoon depicts general ER redistribution of Tango1 (compare to surrounding interphase cells).

(B) Distribution of Sec16 (Sec16-sGFP, green) in wing disc cells undergoing mitosis. Phalloidin staining shown in red (F-actin). DNA stained with DAPI (blue). Top row panels show three examples, while bottom row panels show the same images with the dividing cells outlined in white and without the red (phalloidin) channel. Sec16 puncta disappear (compare to interphase cells).

(C) Distribution of Sec12 (UAS-Sec12-CFP, green) in wing disc cells undergoing mitosis. Phalloidin staining shown in red (F-actin). DNA stained with DAPI (blue). Top row panels show three examples, while bottom row panels show the same images with the dividing cells outlined and without the red (phalloidin) channel. The cartoon depicts general ER distribution of Sec12, same as in interphase cells.

(D) Distribution of Golgin245 (anti-body staining, green) in wing disc cells undergoing mitosis. Phalloidin staining shown in red (F-actin). DNA stained with DAPI (blue). Top row panels show three examples, while bottom row panels show the same images with the dividing cells outlined in white and without the red (phalloidin) channel. The cartoon depicts general punctate Golgi signal, same as in interphase cells.

Journal of Cell Science • Supplementary information

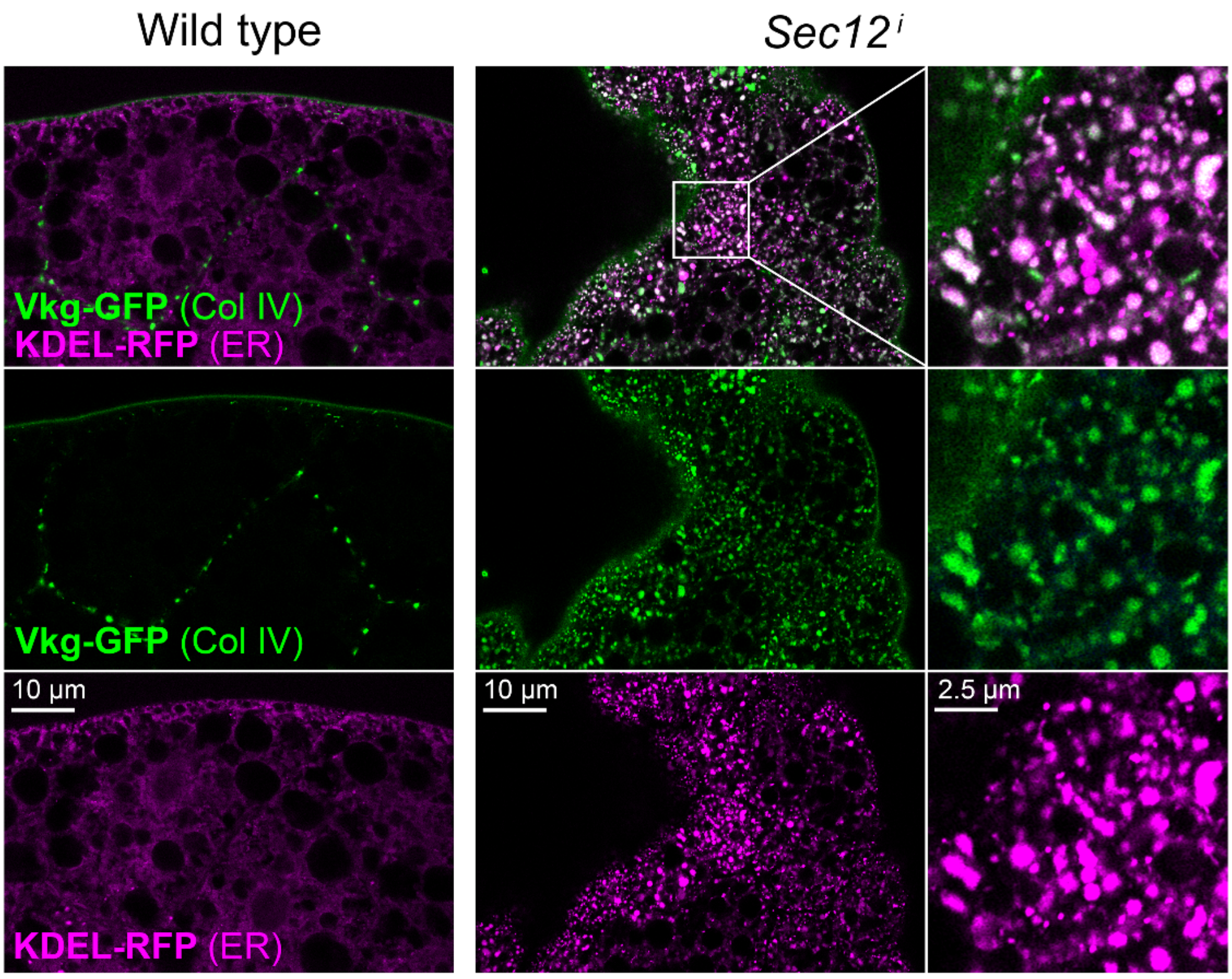

Fig. S3. Sec12 loss causes retention of secretory cargo inside the ER

Distribution of Collagen IV (endogenous Vkg-GFP, green) in wild type fat body (left) and fat body where Sec12 has been knocked down (right). Collagen IV intracellularly retained upon Sec12 knock down localizes to rounded structures containing the ER marker KDEL-RFP (magenta).

Journal of Cell Science • Supplementary information

Figure 5D

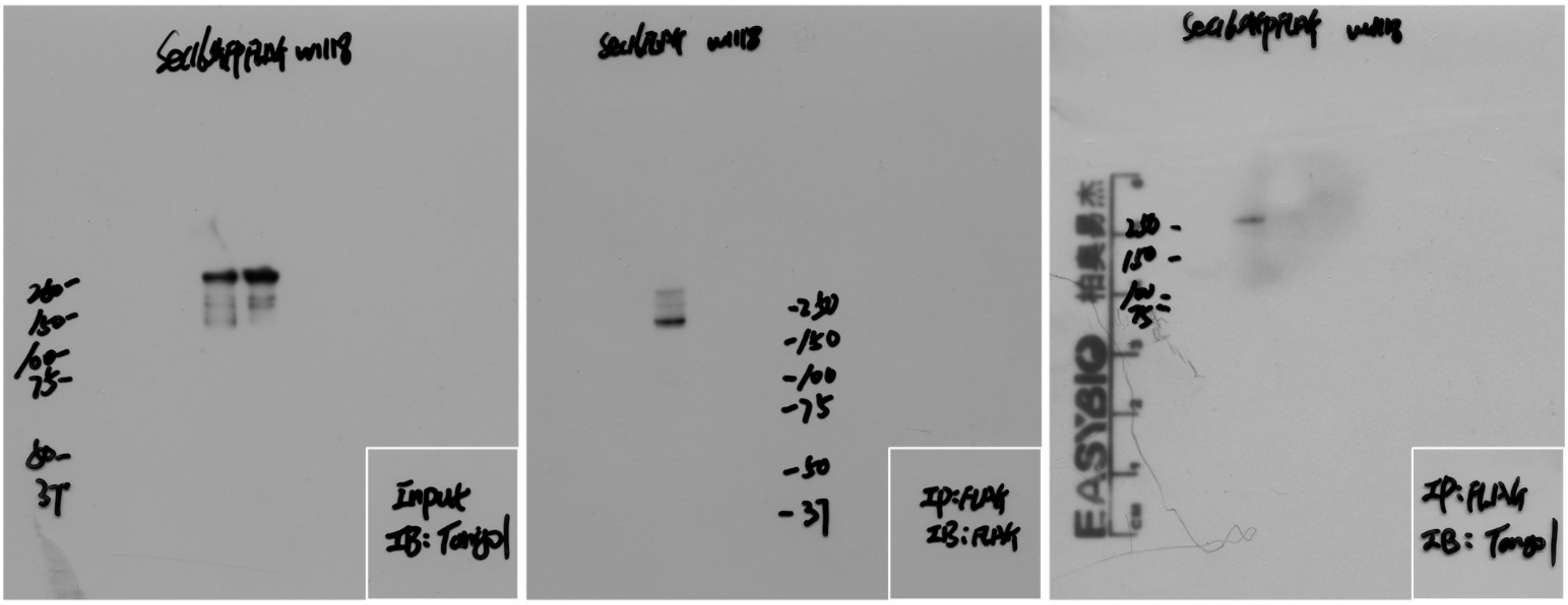

Figure 5E

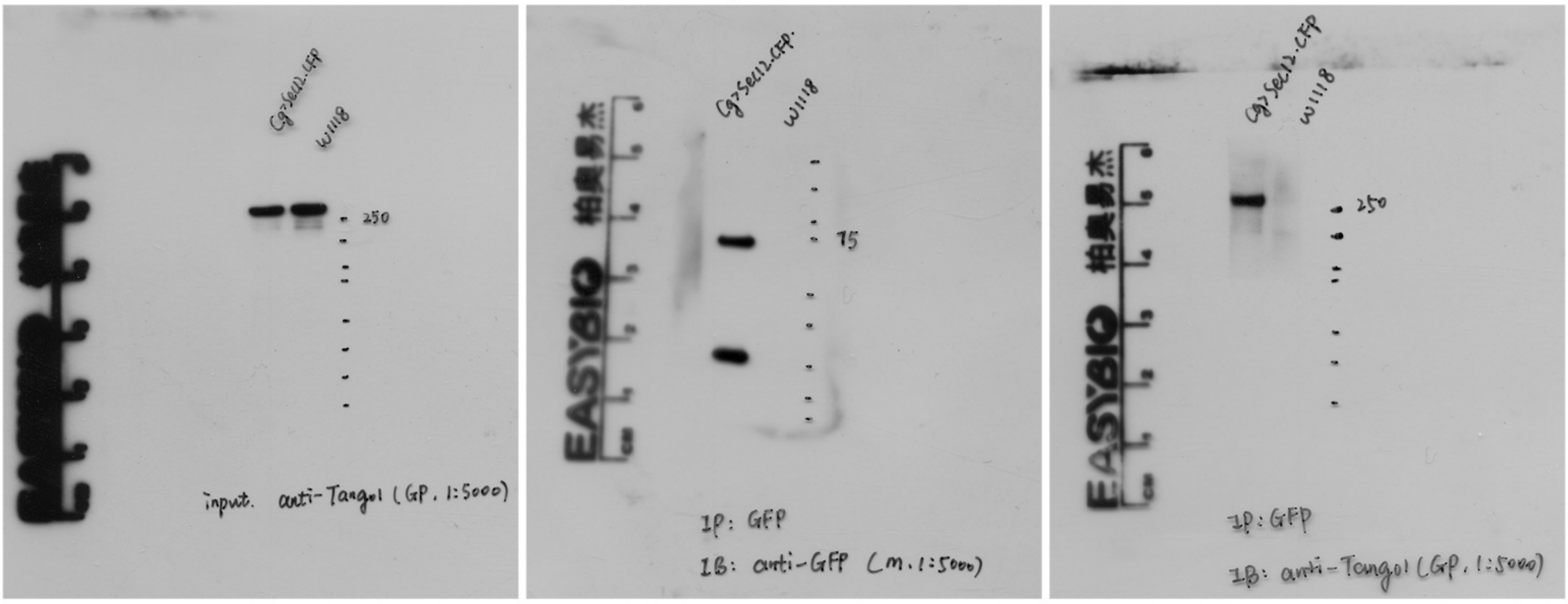

Fig. S4. Blot transparency

Uncropped Western immunoblots in Figure 5D and E.

Journal of Cell Science • Supplementary information

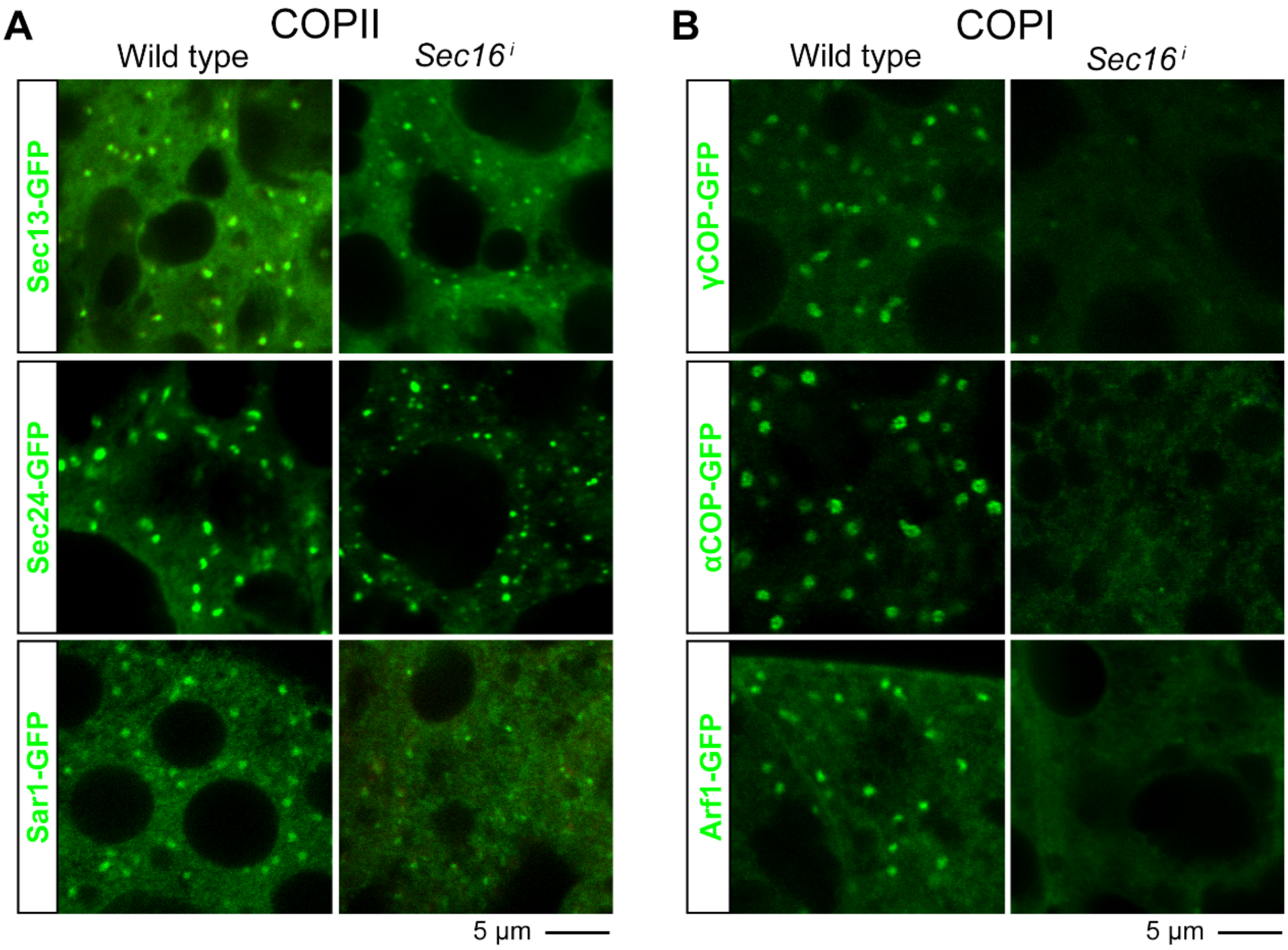

Fig. S5. Loss of Sec16 differentially affects recruitment of COPI to ERES

(A) Distribution in wild type (left column) and *Sec16*^{i} (right column) fat body of COPII coat proteins Sec13 (UAS-Sec13-GFP, green; top row) and Sec24 (UAS-Sec24-GFP, green; center row), as well as COPII GTPase Sar1 (UAS-Sar1-GFP, green; bottom row). Notice that puncta in *Sec16*^{i} are smaller but readily discernible.

(B) Distribution in wild type (left column) and *Sec16*^{i} (right column) fat body of COPI coat proteins γCOP (UAS- γCOP-GFP, green; top row) and αCOP (UAS-αCOP-GFP, green; center row), as well as COPI GTPase Arf1 (UAS-Arf79F-GFP, green; bottom row). Notice no puncta in *Sec16*^{i} are apparent.

Journal of Cell Science • Supplementary information

Table S1. Experimental genotypes

**Figure 1. Mutual recruitment of Tango1 and Sec16 to ERES**	
**B**	*w ; Cg-GAL4 / + ; Tango1-sGFP*^{fTRG00602} / +
	w ; Cg-GAL4 / UAS-RFP-Sec16 ; Tango1-sGFP^{fTRG00602} / +
**C**	*w ; Cg-GAL4 / + ; Sec16-sGFP*^{fTRG01259} / +
	w ; Cg-GAL4 / UAS-Tango1-FLAG ; Sec16-sGFP^{fTRG01259} / +
**D**	*w ; Cg-GAL4 / UAS-RFP-Sec16*
**E**	*w ; Cg-GAL4 / + ; UAS-SP-GFP-Tango1 / +*
**F**	*w ; Cg-GAL4 / UAS-RFP-Sec16 ; UAS-SP-GFP-Tango1 / +*
**G, H**	*w ; Cg-GAL4 / + ; Sec16-sGFP*^{fTRG01259} / +
	w ; Cg-GAL4 / UAS-RFP-Sec16
	w ; Cg-GAL4 / + ; UAS-SP-GFP-Tango1 / +
	w ; Cg-GAL4 / UAS-RFP-Sec16 ; UAS-SP-GFP-Tango1 / +

**Figure 2. Loss of Tango1 or Sec16 causes reduction of ERES size**	
**A**	*w ; Cg-GAL4 / + ; Tango1-sGFP*^{fTRG00602} / +
	w / y v OR Y *; Cg-GAL4 / UAS-Sec16.RNAi*$^{TRiP.HMJ21252}$ *; Tango1-sGFP*^{fTRG00602} / +
**B**	*w ; Cg-GAL4 / + ; Sec16-sGFP*^{fTRG01259} / +
	w ; Cg-GAL4 / + ; Sec16-sGFP^{ffTRG01259} / *UAS-Tango1.RNAi*$^{NIG.11098R\text{-}3}$
**C**	*w ; Cg-GAL4 / + ; Sec16-sGFP*^{fTTRG01259} / +
**D**	*w ; Cg-GAL4 / + ; Sec16-sGFP*^{fTRG01259} / +
	w ; Cg-GAL4 / + ; Sec16-sGFP^{fTRG01259} / *UAS-Tango1.RNAi*$^{NIG.11098R\text{-}3}$
**E**	*w ; Cg-GAL4 / + ; Tango1-sGFP*^{fTRG00602} / +
**F**	*w ; Cg-GAL4 / + ; Tango1-sGFP*^{fTRG00602} / +
	w / y v OR Y *; Cg-GAL4 / UAS-Sec16.RNAi*$^{TRiP.HMJ21252}$ *; Tango1-sGFP*^{fTRG00602} / +
**G, H**	*w ; Cg-GAL4 / + ; Tango1-sGFP*^{fTRG00602} / +
	w / y v OR Y *; Cg-GAL4 / UAS-Sec16.RNAi*$^{TRiP.HMJ21252}$ *; Tango1-sGFP*^{fTRG00602} / +
**I, J**	*w ; Cg-GAL4 / + ; Sec16-sGFP*^{fTRG01259} / +
	w ; Cg-GAL4 / + ; Sec16-sGFP^{ffTRG01259} / *UAS-Tango1.RNAi*$^{NIG.11098R\text{-}3}$

**Figure 3. *Drosophila* Sec12 encoded by *CG9175* is required for secretion**	
**C**	*w ; Cg-GAL4 / + ; UAS-Sec12-CFP / UAS-tdTom-Sec61β*
**D**	*w ; UAS-secr-GFP / + ; BM-40-SPARC-GAL4 / +*
	w / y sc v OR Y *; UAS-secr-GFP / UAS-Sec12.RNAi*^{FZ1} *; BM-40-SPARC-GAL4 / +*
	w / y sc v OR Y *; UAS-secr-GFP / UAS-Sec12.RNAi*^{FZ2} *; BM-40-SPARC-GAL4 / +*
**E**	*w ; vkg*^{G454}*-GFP / + ; BM-40-SPARC-GAL4 / +*
	w / y sc v OR Y *; vkg*^{G454}*-GFP / UAS-Sec12.RNAi*^{FZ1} *; BM-40-SPARC-GAL4 / +*
	w / y sc v OR Y *; vkg*^{G454}*-GFP / UAS-Sec12.RNAi*^{FZ2} *; BM-40-SPARC-GAL4 / +*

**Figure 4. Loss of Sec12 uncouples Sec16 and Tango1**	
**A-E**	*w ; Cg-GAL4 / + ; Sec16-sGFP*^{fTRG01259} / +
	w / y sc v OR Y *; Cg-GAL4 / UAS-Sec12.RNAi*^{FZ1} *; Sec16-sGFP*^{fTRG01259} / +

Journal of Cell Science • Supplementary information

**Figure 5. *Drosophila* Tango1 interacts with Sec12 and Sec16**	
**A**	*w ; Cg-GAL4 / + ; UAS-Sec12-CFP / +*
	w ; Cg-GAL4 / + ; UAS-Sec12-CFP / UAS-RFP-Sec16
	w ; Cg-GAL4 / + ; UAS-Sec12-CFP / UAS-SP-GFP-Tango1
**B**	*w ; Cg-GAL4 / UAS-Tango1-FLAG ; UAS-Sec12-CFP / UAS-tdTom-Sec61β*
**C**	*w ; Cg-GAL4 / UAS-Tango1-FLAG ; UAS-Sec12-CFP / UAS-RFP-Sec16*
**D**	w^{1118}
	y w ; Sec16-sGFP^{fTRG01259}
**E**	w^{1118}
	w ; Cg-GAL4 / + ; UAS-Sec12-CFP / +

**Figure 6. ERES-Golgi coupling depends on Tango1, but not Sec16**	
**A**	*w ; Cg-GAL4 / + ; Sec16-sGFP*^{fTRG01259} */ +*
	w ; Cg-GAL4 / + ; Sec16-sGFP^{fTRG01259} */ UAS-Tango1.RNAi*$^{NIG.11098R-3}$
**B**	*w ; Cg-GAL4 / + ; Tango1-sGFP*^{fTRG00602} */ +*
	w / y v OR Y *; Cg-GAL4 / UAS-Sec16.RNAi*$^{TRiP.HMJ21252}$ *; Tango1-sGFP*^{fTRG00602} */ +*
**C**	*w ; Cg-GAL4 / + ; Sec16-sGFP*^{fTRG01259} */ +*
	w ; Cg-GAL4 / + ; Sec16-sGFP^{fTRG01259} */ UAS-Tango1.RNAi*$^{NIG.11098R-3}$
**D**	*w ; Cg-GAL4 / + ; Tango1-sGFP*^{fTRG00602} */ +*
	w / y v OR Y *; Cg-GAL4 / UAS-Sec16.RNAi*$^{TRiP.HMJ21252}$ *; Tango1-sGFP*^{fTRG00602} */ +*
**E**	*w ; Cg-GAL4 vkg*^{G454}*-GFP / + ; UAS-Tango1.RNAi*$^{NIG.11098R-3}$ */ +*
**F**	*w ; Cg-GAL4 vkg*^{G454}*-GFP / UAS-Sec16.RNAi*$^{TRiP.HMJ21252}$

**Figure 7. Effects of Tango1 and Sec16 loss on recruitment of COPII and COPI to ERES**	
**A, B**	*w ; Cg-GAL4 / + ; UAS-Sec13-EGFP-APEX2 UAS-γCOP-mRFP / +*
	w ; Cg-GAL4 / + ; UAS-Sec13-EGFP-APEX2 UAS-γCOP-mRFP / UAS-Tango1.RNAi$^{NIG.11098R-3}$
**C, D**	*w ; Cg-GAL4 / + ; UAS-Sec13-EGFP-APEX2 UAS-γCOP-mRFP / +*
	w ; Cg-GAL4 / UAS-Sec16.RNAi$^{TRiP.HMJ21252}$ *; UAS-Sec13-EGFP-APEX2 UAS-γCOP-mRFP / +*

**Figure S1. Combined effects of Sec16 overexpression with Tango1 knock down and vice versa**	
**A**	*w ; Cg-GAL4 UAS-RFP-Sec16 / +*
**B**	*w ; Cg-GAL4 UAS-RFP-Sec16 / + ; UAS-Tango1.RNAi*$^{NIG.11098R-3}$ */ +*
**C**	*w ; Cg-GAL4 UAS-SP-GFP-Tango1 / +*
**D**	*w ; Cg-GAL4 UAS-SP-GFP-Tango1 / + ; UAS-Sec16.RNAi*$^{TRiP.HMJ21252}$ */ +*
**E, F**	*w ; Cg-GAL4 UAS-RFP-Sec16 / +*
	w ; Cg-GAL4 UAS-RFP-Sec16 / + ; UAS-Tango1.RNAi$^{NIG.11098R-3}$ */ +*
	w ; Cg-GAL4 UAS-SP-GFP-Tango1 / +
	w ; Cg-GAL4 UAS-SP-GFP-Tango1 / + ; UAS-Sec16.RNAi$^{TRiP.HMJ21252}$ */ +*

**Figure S2. Distribution of Tango1, Sec16 and Sec12 in dividing cells**	
**A**	*y w ; Tango1-sGFP*^{fTRG00602}
**B**	*y w ; Sec16-sGFP*^{fTRG01259}
**C**	*w ; rn-GAL4 / UAS-Sec12-CFP*
**D**	w^{1118}

**Figure S3. Loss of Sec12 causes retention of secretory cargo inside the ER**	
	w ; Cg-GAL4 vkg^{G454}-GFP / + ; UAS-RFP-KDEL / +
	w ; Cg-GAL4 vkg^{G454}-GFP / UAS-Sec12.RNAi^{FZ1} ; UAS-RFP-KDEL / +

**Figure S4. Blot transparency**	
	w^{1118}
	y w ; Sec16-sGFP^{fTRG01259}
	w ; Cg-GAL4 / + ; UAS-Sec12-CFP / +

**Figure S5. Loss of Sec16 differentially affects recruitment of COPI to ERES**	
A	w ; Cg-GAL4 / + ; UAS-Sec13-EGFP-APEX2 / +
	w ; Cg-GAL4 / UAS-Sec16.RNAi$^{TRiP.HMJ21252}$; UAS-Sec13-EGFP-APEX2 / +
	w ; Cg-GAL4 / + ; UAS-EGFP-APEX2-Sec24CD(PC) / +
	w ; Cg-GAL4 / UAS-Sec16.RNAi$^{TRiP.HMJ21252}$; UAS-EGFP-APEX2-Sec24CD(PC) / +
	w ; Cg-GAL4 / + ; Sar1^{CA07674}-GFP / +
	w ; Cg-GAL4 / UAS-Sec16.RNAi$^{TRiP.HMJ21252}$; Sar1^{CA07674}-GFP / +
B	w ; Cg-GAL4 / + ; UAS-γCOP-APEX2-EGFP / +
	w ; Cg-GAL4 / UAS-Sec16.RNAi$^{TRiP.HMJ21252}$; UAS-γCOP-APEX2-EGFP / +
	w ; Cg-GAL4 / + ; αCOP-sGFP^{fTRG01193} / +
	w ; Cg-GAL4 / UAS-Sec16.RNAi$^{TRiP.HMJ21252}$; αCOP-sGFP^{fTRG01193} / +
	w ; Cg-GAL4 / + ; UAS-Arf1-EGFP-APEX2 / +
	w ; Cg-GAL4 / UAS-Sec16.RNAi$^{TRiP.HMJ21252}$; UAS-Arf1-EGFP-APEX2 / +
