# Supplementary material for: Cooperative definition of ER exit sites by Tango1, Sec16 and Sec12
Source: J Cell Sci. 2026 Sep 29;139(21):jcs264950. doi: 10.1242/jcs.264950 (PMC13723486; doi:10.1242/jcs.264950)
Supplement: Supplementary file 2 [file joces-139-264950_review_history.pdf]

Journal of Cell Science

Cooperative definition of ER exit sites by Tango1, Sec16 and Sec12

Zhi Feng, Mercedes Martín, Marta Rojas and José Carlos Pastor-Pareja

DOI: 10.1242/jcs.264950

Editor: Giulia Zanetti

Review timeline

Original submission:	7 April 2026
Editorial decision:	30 April 2026
First revision received:	24 July 2026
Accepted:	24 July 2026

Original submission

First decision letter

MS ID#: jcs.264950

MS TITLE: Cooperative definition of ER exit sites by Tango1, Sec16 and Sec12

AUTHORS: Zhi Feng; Mercedes Martín; José Carlos Pastor-Pareja

ARTICLE TYPE: Research Article

Dear Dr Pastor-Pareja,

We have now reached a decision on the above manuscript.

As you will see, the reviewers gave favourable reports but raised some critical points that will require amendments to your manuscript. I hope that you will be able to carry these out because I would like to be able to accept your paper, depending on further comments from reviewers.

Reviewer 1

TANGO1, Sec16, Sec12, COPS are well known proteins that function in the secretory pathway. The authors provide convincing data on the mutual impact of these proteins at the ERES-Golgi junction. There is nothing really wrong with the data. This would appeal to the experts, but one way to make it really impressive is to provide CLEM based EM data on the organisation of the wild type and mutant ERES that are either depleted of TANGO1, Sec16 and cells that over express these proteins. But this is really asking for a lot and might not be possible at this stage. SO, I leave it to the authors to decide. One other point, that might be much easier to address, is to test how are COPI proteins interacting with the ERES components? This would bring something new to the understanding of how ERES and COPI communicate to ensure cargo traffic at the ERES-Golgi junction. But I would be happy to go without this data, if it is too technically challenge.

Reviewer 2

SUMMARY OF THE ADVANCE MADE IN THIS PAPER AND ITS POTENTIAL SIGNIFICANCE TO THE FIELD

© 2026. Published by The Company of Biologists under the terms of the Creative Commons Attribution License (https://creativecommons.org/licenses/by/4.0/).

This well-executed study documents the interdependent localisations of conserved ERES proteins, Sec16, Tango-1 and Sec12 in a Drosophila model system. The findings add to our understanding of how ERES are organised and establish the likely Drosophila ortholog of Sec12, which was previously uncharacterized. Although largely descriptive, the imaging is clear and robust, and this is a nice contribution to the field, which currently debates the functions of these proteins.

SUGGESTIONS TO AUTHORS

I have only minor comments:

1. How do ERES look in TANGO-OE and SEC16-OE conditions when the opposing partner is knocked down? Are ERES able to be remodelled by TANGO and SEC16 alone? This should be an easy experiment: OE TANGO/SECC16 in the SEC16/TANGO KD background.

2. In Figure 3D-E including an ER marker would be helpful. I assume the odd structures that contain cargo are dilated ER but this should be confirmed.

3. In Figure 4, there seems to be a missed opportunity to quantify more aspects of the ERES: Manders coefficient for co-location; number of punch, size of puncta. This is a data-rich experiment that could reveal more.

First revision

Author response to reviewers' comments

Response to reviewers

We wish to thank the two expert referees for carefully reviewing our manuscript, for their positive appreciation of our work and for their suggestions.

Reviewer 1:

TANGO1, Sec16, Sec12, COPS are well known proteins that function in the secretory pathway. The authors provide convincing data on the mutual impact of these proteins at the ERES-Golgi junction. There is nothing really wrong with the data. This would appeal to the experts, but one way to make it really impressive is to provide CLEM based EM data on the organisation of the wild type and mutant ERES that are either depleted of TANGO1, Sec16 and cells that over express these proteins. But this is really asking for a lot and might not be possible at this stage. So, I leave it to the authors to decide.

We thank the reviewer for the suggestion to document ERES organization in wild type and mutant conditions at electron microscopy (EM) resolution. We have previously performed similar analyses for Tango1 overexpression (Yang et al., 2021) and p24 mutants (Yang et al., 2024) using FIB-SEM without the need for CLEM, as we find that ERES can be reliably identified in *Drosophila* cells based on ultrastructural features alone.

We fully agree that a high-resolution characterization of additional mutant conditions, such as *Sec16* loss- and gain-of-function, *Tango1* loss-of-function, and *Sec12* loss-of-function, would provide valuable information. However, carrying out these analyses would require a substantial experimental effort and would considerably broaden the scope of the manuscript. We therefore believe that such work is better suited for a future study rather than to the current manuscript, particularly within the time frame available for its revision.

One other point, that might be much easier to address, is to test how are COPI proteins interacting with the ERES components? This would bring something new to the understanding of how ERES and COPI communicate to ensure cargo traffic at the ERES-Golgi junction. But I

© 2026. Published by The Company of Biologists under the terms of the Creative Commons Attribution License (https://creativecommons.org/licenses/by/4.0/).

would be happy to go without this data, if it is too technically challenge.

We appreciate the reviewer's suggestion to address the relationship between COPI and ERES components and fully agree that this is a particularly interesting question. In contrast to the interactions between ERES proteins and the COPII machinery, which have been extensively characterized and are considered fundamental for ERES organization and function, the molecular links between COPI and ERES components remain largely unexplored. The close spatial association between COPI and ERES observed here and in other studies raises the possibility that direct molecular interactions may exist. Establishing these interactions conclusively would require extensive follow-up work, including the mapping of the interacting domains and residues through targeted mutagenesis. We believe that these experiments constitute a substantial research project in its own right and would considerably broaden the scope of the manuscript. They are therefore better suited for a dedicated future study. Nevertheless, we have revised the Discussion to better emphasize the potential importance of the ERES-COPI relationship and to contrast it with the better understood interactions between ERES components and the COPII machinery, as follows:

Line 348: In contrast to the interactions between ERES proteins and the COPII machinery, which have been characterized and are considered central to ERES assembly and function, possible mechanisms by which Tango1 and Sec16 recruit COPI to ERES remain unexplored. Given the close association of COPI with ERES, where COPI localizes to the periphery of *Drosophila* ERES cups in (Yang et al., 2021), it is tempting to speculate that direct physical interactions between COPI components and these ERES proteins contribute to ERES organization. Defining these interactions will be an important objective for future studies and may provide new insight into how anterograde and retrograde trafficking are coordinated at the ER-Golgi interface.

Reviewer 2:

SUMMARY OF THE ADVANCE MADE IN THIS PAPER AND ITS POTENTIAL SIGNIFICANCE TO THE FIELD

This well-executed study documents the interdependent localisations of conserved ERES proteins, Sec16, Tango-1 and Sec12 in a Drosophila model system. The findings add to our understanding of how ERES are organised and establish the likely Drosophila ortholog of Sec12, which was previously uncharacterized. Although largely descriptive, the imaging is clear and robust, and this is a nice contribution to the field, which currently debates the functions of these proteins.

SUGGESTIONS TO AUTHORS

I have only minor comments:

1. How do ERES look in TANGO-OE and SEC16-OE conditions when the opposing partner is knocked down? Are ERES able to be remodelled by TANGO and SEC16 alone? This should be an easy experiment: OE TANGO/SEC16 in the SEC16/TANGO KD background.

We thank the reviewer for this suggestion to further investigate the relationship between Tango1 and Sec16. We have performed the two proposed experiments by analyzing the effects of Tango1 knockdown in the context of Sec16 overexpression, and conversely, Sec16 knockdown in the context of Tango1 overexpression. Interestingly, in both experimental conditions, the number of ERES is reduced, resembling the corresponding loss-of-function phenotype, whereas ERES size is increased, resembling the corresponding overexpression phenotype. We believe these findings provide additional insight into the interplay between Tango1 and Sec16, and we have now included these data in Supplementary Figure S1 and describe them in the Results section.

Line 168: To additionally test the requirement of Tango1 and Sec16 to form ERES, we conducted experiments in which we knocked down the expression of Tango1 or Sec16 while overexpressing the other protein: *Sec16*^{i} + Tango1 OE (Fig. S1A and B) and *Tango1*^{i} + Sec16 OE (Fig. S1C and D). In both cases, we observed a reduction in the number of ERES (Fig. S1E) accompanied by an increase

© 2026. Published by The Company of Biologists under the terms of the Creative Commons Attribution License (https://creativecommons.org/licenses/by/4.0/).

in average puncta size (Fig. S1F), indicating phenotypic prevalence of the knock down and overexpression conditions on ERES number and ERES size, respectively.

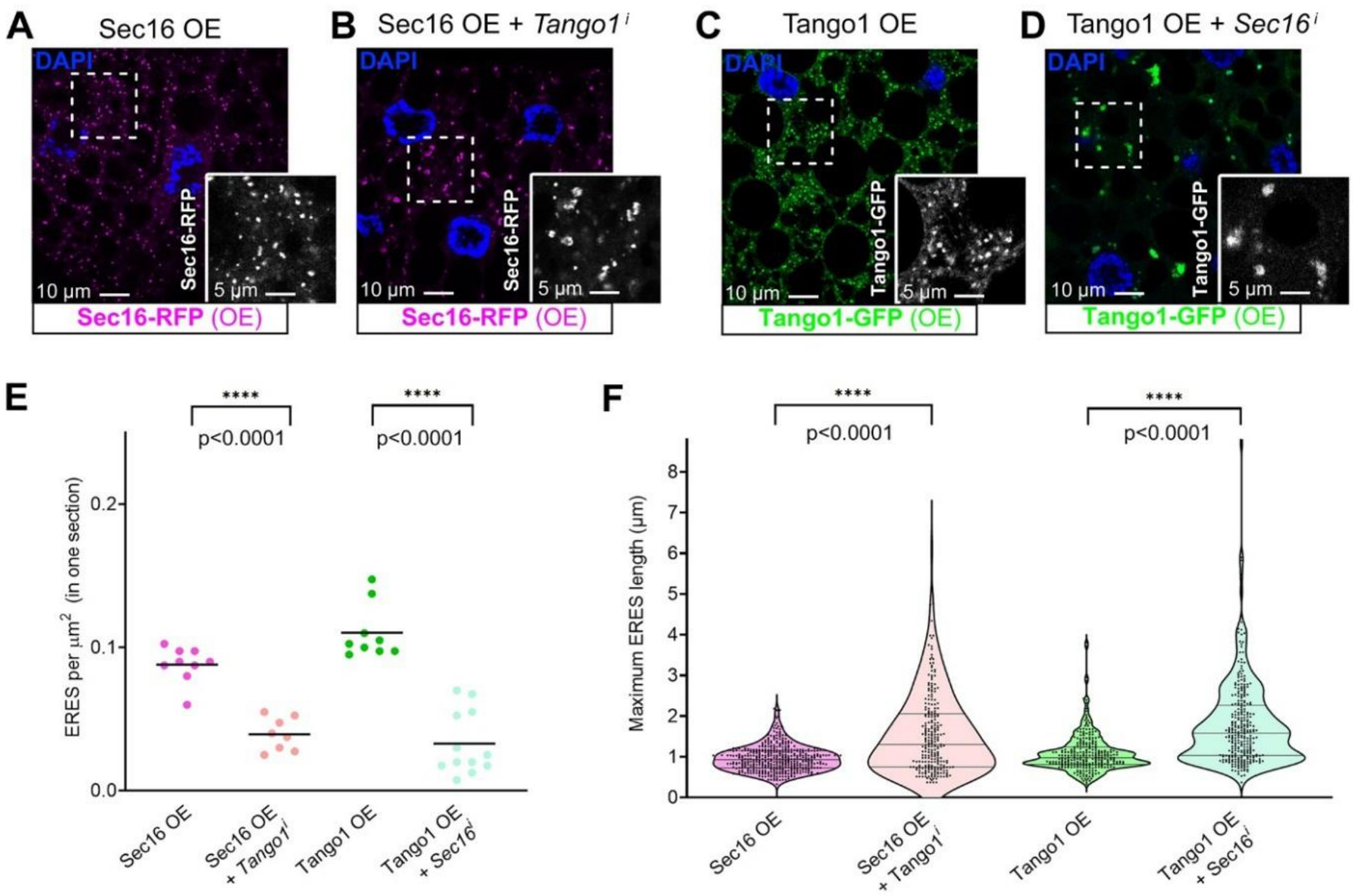

Figure S1. Combined effects of Sec16 overexpression with Tango1 knock down and vice versa

(A) Distribution of overexpressed Sec16 (UAS-Sec16-RFP, magenta) in fat body adipocytes. Nuclei stained with DAPI (blue). Image in bottom right corner (Sec16-RFP, white) shows magnified view of inset (dashed square).

(B) Distribution of overexpressed Sec16 (UAS-Sec16-RFP, magenta) in fat body where Tango1 has been knocked down (Sec16 OE + *Tango1^{i}*). Image in bottom right corner (Sec16-RFP, white) shows magnified view of inset (dashed square).

(C) Distribution of overexpressed Tango1 (UAS-SP-GFP-Tango1, green) in fat body adipocytes. Nuclei stained with DAPI (blue). Image in bottom right corner (Tango1-GFP, white) shows magnified view of inset (dashed square).

(D) Distribution of overexpressed Tango1 (UAS-SP-GFP-Tango1, green) in fat body where Sec16 has been knocked down (Tango1 OE + *Sec16^{i}*). Image in bottom right corner (Tango1-GFP, white) shows magnified view of inset (dashed square).

(E) Graph comparing ERES abundance in Sec16 OE, Sec16 OE + *Tango1^{i}*, Tango1 OE and Tango1 OE + *Sec16^{i}* fat body adipocytes. Each dot represents a measurement of the density of puncta counted inside a square of the dimensions of the insets in A-D. n=10. Means were significantly different in two-tailed unpaired t tests, with p values reported in the graph.

(F) Graph comparing ERES size in Sec16 OE, Sec16 OE + *Tango1^{i}*, Tango1 OE and Tango1 OE + *Sec16^{i}*. fat body adipocytes. Each dot represents a measurement of the maximum length of a punctum. n≥245. Violin plots depict the median value and interquartile range. Significance of differences was determined through two-tailed unpaired t tests, with p values reported in the graph.

2. In Figure 3D-E including an ER marker would be helpful. I assume the odd structures that

© 2026. Published by The Company of Biologists under the terms of the Creative Commons Attribution License (https://creativecommons.org/licenses/by/4.0/).

contain cargo are dilated ER but this should be confirmed.

We thank the reviewer for this helpful suggestion. To confirm the location of the accumulated secretory cargo upon Sec12 loss, we repeated the experiment including the ER marker KDEL-RFP. As expected, the intracellularly retained secretory cargo (Collagen IV) colocalizes with the ER marker, confirming that cargo accumulation occurs within the ER and is accompanied by ER dilation. These new data support our initial interpretation of the phenotype and are now included in Supplementary Figure S2.

Line 209: This intracellularly retained Collagen IV localized to rounded structures positive for the ER marker KDEL-RFP (Fig. S2), indicating that retention took place inside dilated ER cisternae.

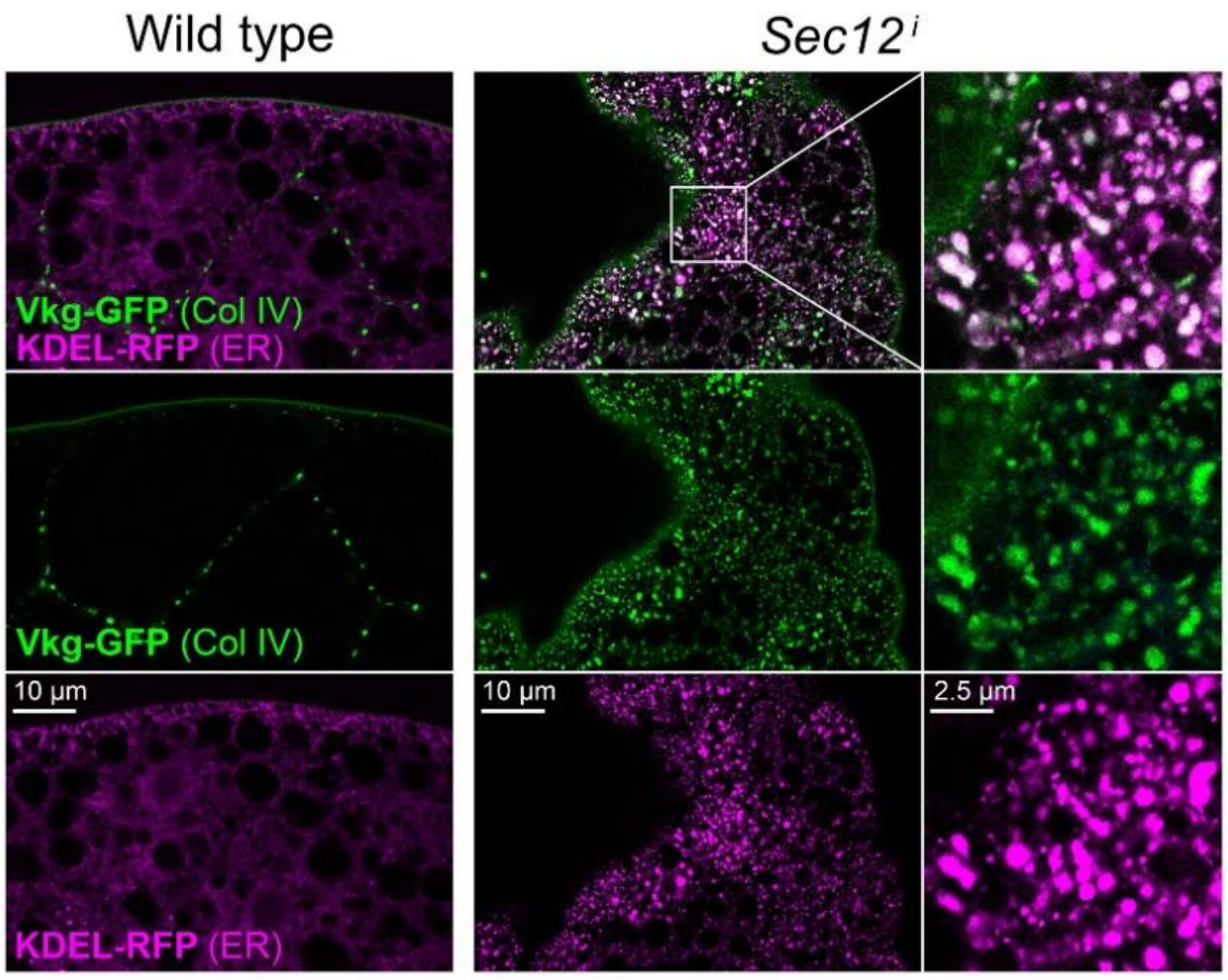

Figure S2. Sec12 loss causes retention of secretory cargo inside the ER

Distribution of Collagen IV (endogenous Vkg-GFP, green) in wild type fat body (left) and fat body where Sec12 has been knocked down (right). Collagen IV intracellularly retained upon Sec12 knock down localizes to rounded structures containing the ER marker KDEL-RFP (magenta).

3. In Figure 4, there seems to be a missed opportunity to quantify more aspects of the ERES: Manders coefficient for co-location; number of puncta, size of puncta. This is a data-rich experiment that could reveal more.

We thank the reviewer for this valuable suggestion to further quantify the Sec12 loss-of-function phenotype. In addition to assessing the dissociation of Tango1 and Sec16 puncta, we have now performed a more comprehensive quantitative analysis. Specifically, we quantified the degree of colocalization between Tango1 and Sec16 using both Manders' overlap coefficients and Pearson's correlation coefficient. We also measured the number and size of puncta, distinguishing between puncta containing only Tango1, only Sec16, or both proteins in the Sec12 loss-of-function condition. These additional analyses provide a more complete characterization of the phenotype and are now presented in graphs in Figure 4C-E.

Line 217: Quantification using Pearson’s correlation and Manders’ overlap coefficients confirmed reduced Ta ngo1-Sec16 colocalization, consistent with their dissociation upon Sec12 knock down (Fig. 4C). Furthermore, the size of puncta containing only Tango1 or only Sec16 was significantly reduced compared with the wild type, whereas the size of puncta retaining both proteins was not significantly affected (Fig. 4D). Finally, quantification of puncta abundance following Sec12 depletion revealed no significant difference in the total number of puncta relative to the wild type when puncta containing only Tango1, only Sec16, or both proteins were considered together (Fig. 4E).

© 2026. Published by The Company of Biologists under the terms of the Creative Commons Attribution License (https://creativecommons.org/licenses/by/4.0/).

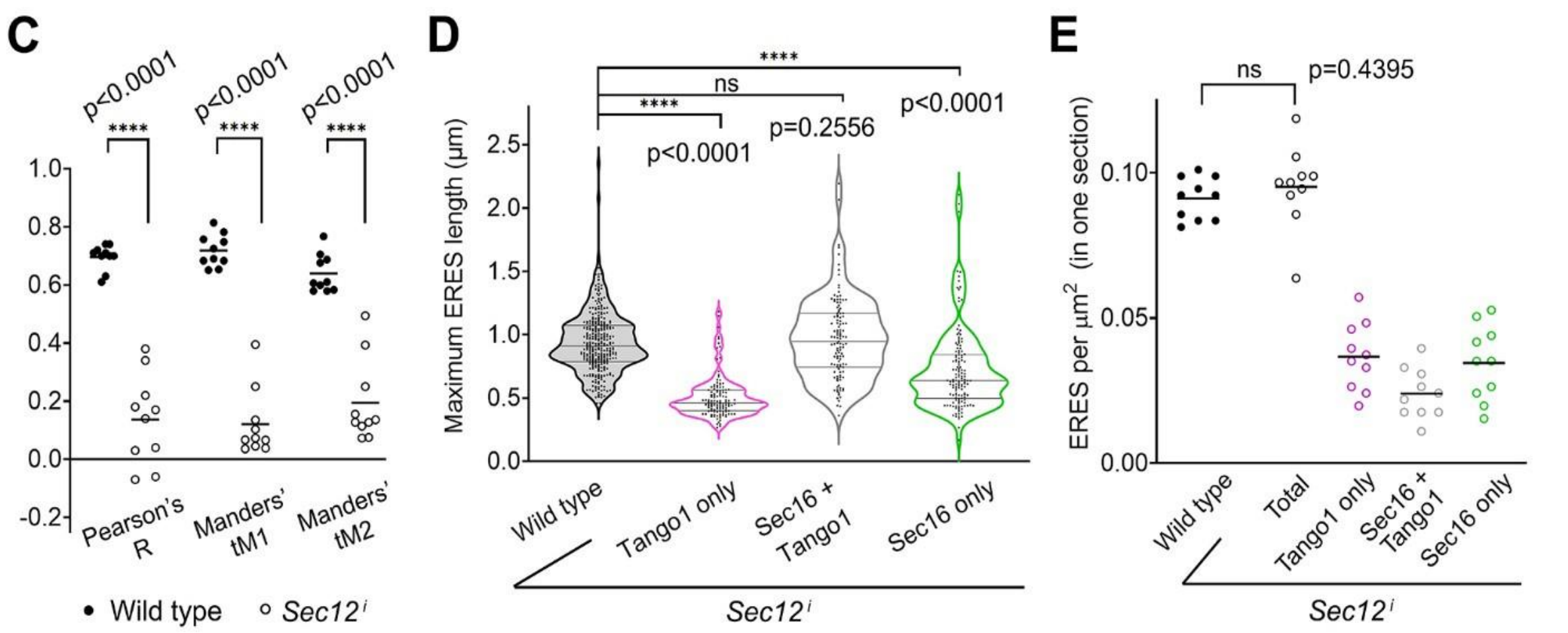

Figure 4. Loss of Sec12 uncouples Sec16 and Tango1

(C) Graph quantifying Tango1-Sec16 colocalization through Pearson's and Manders' coefficients after Costes thresholding in wild type and *Sec12^{i}* individual fat body adipocytes. Means were significantly different from the wild type in two-tailed unpaired t tests, with p values reported in the graph.

(D) Graph comparing ERES size in wild type and *Sec12^{i}* fat body adipocytes. Each dot represents a measurement of the maximum length of a punctum. n≥119. Violin plots depict the median value and interquartile range. Significance of differences of means was determined through two-tailed unpaired t tests, with p values reported in the graph.

(E) Graph comparing ERES abundance in wild type and *Sec12^{i}* fat body adipocytes. Each dot represents a measurement of the density of puncta counted inside a square of the dimensions of the insets in A and E. n=10. Means were significantly different in a two-tailed unpaired t test, with p values reported in the graph.

Second decision letter

MS ID#: jcs.264950R1

MS Title: Cooperative definition of ER exit sites by Tango1, Sec16 and Sec12

Authors: Zhi Feng; Mercedes Martín; Marta Rojas; José Carlos Pastor-Pareja
Article Type: Research Article

Dear Dr Pastor-Pareja,

thank you for submitting a revised version of the manuscript. Your revisions are satisfactory and I do not need to send the manuscript back to reviewers. I am therefore happy to tell you that your manuscript has been accepted for publication in Journal of Cell Science, pending standard publication integrity checks.

© 2026. Published by The Company of Biologists under the terms of the Creative Commons Attribution License (https://creativecommons.org/licenses/by/4.0/).
